# Impact of fresh and fermented vegetable consumption on gut microbiota and body composition: insights from diverse data analysis approaches

**DOI:** 10.3389/fnut.2025.1623710

**Published:** 2025-07-15

**Authors:** Susan Pihelgas, Kristel Ehala-Aleksejev, Mari-Liis Kutti, Rain Kuldjärv, Jekaterina Kazantseva

**Affiliations:** ^1^AS TFTAK, Tallinn, Estonia; ^2^School of Natural Sciences and Health, Tallinn University, Tallinn, Estonia

**Keywords:** fermented vegetables, kimchi, gut microbiota, body composition, 16S rRNA amplicon sequencing, nutritional intervention study

## Abstract

**Background:**

The impact of fermented foods on human health, particularly through gut microbiota, is a widely discussed topic. However, the number of nutritional studies demonstrating their health benefits remains limited. This study evaluated the effects of fermented vegetables (FVs) intake including fermented carrots, kohlrabi, and kimchi on human health parameters, with a primary focus on gut microbiota. In addition to microbiota analysis, we assessed anthropometric parameters, stool frequency, and data from nutritional diaries. A total of 65 volunteers were recruited, of whom 55 completed the study. FVs are valuable health products, combining dietary fibers and lactic acid bacteria, both essential for supporting normal gut microbiota. To better understand the effects of FV consumption in different populations, participants were divided into three groups: controls without reported health problems (CTRL), individuals with constipation (CONS), and those recovering from an antibiotic course (AB). Multiple analytical approaches were applied to evaluate: (1) the effects of FV consumption within the three focus groups, (2) the response of different gut enterotypes to a three-week FV consumption period based on taxonomic hierarchical analyses, and (3) the combined effects across the entire cohort.

**Results:**

The choice of analyzed groups was crucial for interpreting the results, revealing varied effects depending on the context. Overall, the findings showed that consuming FVs modified gut microbiota composition, increasing the abundance of butyrate-producing and anti-inflammatory bacterial species. Additionally, phase angle, a key indicator of cellular health reflecting cell integrity and hydration, showed statistically significant improvement across the cohort, indicating better metabolic health after the intervention. Food diaries further revealed a reduction in sugar consumption among participants, highlighting an additional benefit of enriching diets with fermented foods.

**Conclusions:**

These results demonstrate the clear positive impact of FV consumption on human health, particularly through gut microbiota modulation and metabolic improvements.

## 1 Introduction

The microbiota represents a highly intricate and dynamic ecosystem of trillions of microorganisms that play an essential role in sustaining human health. This microbial community exerts a profound influence on critical physiological processes, including digestion, immune system regulation, and metabolic homeostasis, with its balance being closely tied to overall health outcomes (1). The human microbiota begins to develop early in life, undergoing progressive changes that stabilize around the age of three, ultimately forming a composition unique to each individual (2). The microbiota's composition varies markedly across anatomical sites, with each location hosting a specialized microbial community tailored to its specific environmental and physiological context (3, 4). The microbiota found in various body sites are interconnected. For instance, interactions between the oral and intestinal microbiota have been observed, with oral bacteria such as *Fusobacterium* species potentially influencing the gut microbiota which may contribute to intestinal inflammation and related diseases (5, 6). The “mouth-gut axis” refers to the microbial relationship between the mouth and the gut, and research has shown that one in three identifiable salivary microbial cells can colonize the gut, contributing to at least 2% of the identifiable microbial abundance found in feces (7, 8).

Across microbial communities associated with distinct anatomical sites of the human body, the gut microbiota is one of the most diverse, influencing various biological functions, from immune system modulation to behavioral processes. Dysbiosis of the gut microbiota has been associated with various diseases, including gastrointestinal disorders and neurodegenerative conditions. However, defining a healthy gut microbiota remains challenging due to significant individual variability (3, 9). Juline Tap, with his colleagues, conducted a comprehensive analysis across populations and life stages, introducing the theory of branches of the gut microbiome. Their findings suggest that microbiota closer to these branches' central point indicate healthier states (10). Gupta and colleagues (11) developed the Gut Microbiome Health Index (GMHI), a sophisticated predictive taxonomic signature tool for evaluating health status based on the presence of 50 microbial strains associated with health. Complementing this approach, genome-scale models have been employed to link the availability of specific genes to biochemical processes, predicting the implication of gut microbiota for human health (12). This mechanistic model is based on the calculation of the Metabolite Exchange Score (MES), estimating the effects of microbial interactions on clinical outcomes. Together, these methodologies significantly advance our understanding of the role of gut microbiota as a predictive biomarker for health and as a tool for evaluating the impact of dietary changes. However, the field remains vast and largely unexplored, highlighting the need for further research to refine and expand these insights.

Diet is a pivotal determinant of gut microbiota composition (13, 14). A diet rich in dietary fiber fosters the growth of short-chain fatty acid (SCFA)-producing bacteria and reduces gut transit time. Conversely, a Western-style diet, characterized by high-fat and animal-based foods, promotes the growth of bacteria associated with chronic inflammation, increasing the risk of metabolic disorders (15). This underscores the crucial role of dietary patterns in shaping gut microbiota health and mitigating disease risks. Recently published curated Food Metagenomic Data (FMD) have provided clear evidence of the intricate link between food-associated microbial diversity and the human microbiome (16). Notably, this study revealed that up to 3% of the adult gut microbiota comprises food-derived microbes, underscoring the contribution of dietary microbial inputs to the gut ecosystem, striking the dominant influence of habitual dietary patterns in shaping an individual's microbiota composition. These insights further highlight the nuanced interplay between fermented food microbiomes and host gut microbial ecology, offering a new perspective on the role of dietary microbes in health and disease.

Fermented vegetables (FVs) have garnered significant attention for their capacity to modulate the gut microbiota and promote health. Rich in live microorganisms, particularly lactic acid bacteria (LAB), FVs enhance microbial diversity and encourage the proliferation of beneficial gut bacteria (17). Beyond microbiota modulation, they serve as sources of bioactive compounds, dietary fiber, and micronutrients, further amplifying their health benefits (18, 19). Kimchi, a FV product, has been extensively studied for its health-promoting effects. Animal studies demonstrate that kimchi enhances immune function by increasing spleen mass, lymphocyte count, and the expression of IgA, macrophages, and cytokines related to cellular defense (20). A review of 11 studies concluded that kimchi interventions may reduce body weight, alleviate irritable bowel syndrome symptoms, and improve overall health markers (21). Additionally, regular kimchi consumption, even in small amounts, has been shown to lower total cholesterol, low-density lipoprotein levels, and fasting glucose concentrations (22).

Despite promising findings, there remains a paucity of nutritional intervention studies investigating the specific effects of FVs on gut microbiota and health outcomes. Preliminary evidence suggests that individuals with compromised microbiota, such as those experiencing constipation or recent antibiotic use, may benefit significantly from FV consumption. However, the limited number of studies, coupled with variations in methodologies and participant characteristics, highlights the need for further research to establish robust conclusions and personalized dietary recommendations. The present study aimed to investigate the impact of increased consumption of fresh and fermented vegetables on gut microbiota composition and associated health biomarkers. The evaluation was conducted through three distinct analytical approaches: (1) stratifying participants into three predefined groups—controls (CTRL), individuals with constipation (CONS), and individuals recently exposed to antibiotics (AB); (2) clustering participants based on baseline gut microbiota composition; and (3) analyzing the cohort as a whole. Participants underwent a structured multi-week intervention alternating between fresh and fermented vegetable consumption, with a primary focus on characterizing gut microbiota responses and assessing potential health benefits.

## 2 Methods

The current study was carried out, and the recipe for the FVs was developed by AS TFTAK (Center of Food and Fermentation Technologies, Estonia) in collaboration with Kadarbiku Köögivili OÜ (Estonia). All vegetables used in the study were produced by Kadarbiku Köögivili OÜ.

### 2.1 Fresh and fermented vegetables

During the intervention study, participants were asked to consume both fresh and fermented vegetables provided by the organizers. Fresh vegetables included carrot, kohlrabi, and a carrot-cabbage mixture. These products were distributed to participants in three separate portions—one for each week—to ensure freshness, as the shelf life of the fresh vegetables was 10 days. FVs included fermented carrot, fermented kohlrabi, and kimchi. These products were distributed to participants in a single portion intended to last for the entire three-week intervention period, as their shelf life was ~1 month. All fermentation processes were spontaneous and conducted on an industrial scale using traditional method, and special rooms dedicated only to vegetable fermentation. The fermented products were not pasteurized, and participants were instructed to store them at +4°C. Additionally, all participants were advised not to heat or cook the fermented products.

#### 2.1.1 Preparation of fermented vegetables

The vegetable fermentation recipe included 1 kg of fresh vegetables (either kohlrabi or carrot; cut size for both vegetables baton), 10 g of fresh dill, 10 g of garlic, and 1.2 kg of brine. The brine formulation used for the fermentation of vegetables consisted of the following components: 1 l of water, 12 ml of apple cider vinegar (acidity 5%), 35 g of salt, and 20 g of sugar. The fermentation of vegetables was conducted spontaneously over a duration of 5 days at room temperature (20 ± 2°C). The average count of viable microorganisms in the FVs was determined using the standard plating method according to ISO 4833-1:2013 + A1:2022, with a result of 8.3 × 107 CFU/g. The analysis was conducted by the National Center for Laboratory Research and Risk Assessment (LABRIS), Estonia.

#### 2.1.2 Preparation of kimchi

The kimchi recipe included white cabbage, kohlrabi, carrot, soy sauce (water, soybeans, wheat, salt), Korean chili pepper, sugar, salt, ginger and garlic. The white cabbage, kohlrabi, carrots were cut and mixed with paste made from soy sauce, Korean chili pepper, sugar, salt, ginger and garlic. After mixing the fresh kimchi was left to ferment spontaneously for 10 days at room temperature (20 ± 2°C). The average count of viable microorganisms in the kimchi was determined using the standard plating method according to ISO 4833-1:2013 + A1:2022, with a result of 1.7 × 10^8^ CFU/g. The analysis was conducted by the National Center for Laboratory Research and Risk Assessment (LABRIS), Estonia.

### 2.2 Microbial cell separation and genomic DNA extraction

Microbial cells from samples were isolated aseptically under a laminar flow cabinet. First, ~20 ml of fermented vegetable (carrot, kohlrabi and kimchi) liquid was diluted with 20 ml of sterile 0.85% NaCl, vortexed thoroughly and centrifuged at 300 x g for 10 min at 6°C (Hettich ROTANTA 460R, fixed angle rotator) to collect plant debris. To pellet the microbial cells, the supernatant was transferred to a new 50 ml tube and centrifuged at 10,000 x g for 15 min at + 6°C. The resulting pellet was washed in 2 ml of sterile 0.85 % NaCl solution, transferred to a 2 ml tube and centrifuged again at 10,000 x g for 10 min at room temperature (Sigma1-14 microcentrifuge, Sigma Laborzentrifugen GmbH, Germany). The supernatant was aspirated, and the pellet containing the microbial cells were stored at −20°C until genomic DNA (gDNA) extraction.

For gDNA extraction, cells pellets were re-suspended in 250 μl 1 x PBS and subjected to gDNA extraction by ZymoBIOMICS™ DNA Miniprep Kit (Zymo Research, Irvine, CA, USA) according to the manufacturer's instructions. The concentrations of the extracted DNAs were quantified by a Qubit™ 3 Fluorometer (Thermo Fisher Scientific, Waltham, MA, USA) using Qubit dsDNA HS and BR Assay Kits (Thermo Fisher Scientific).

### 2.3 Recruitment of study participants and design of the study

The study was conducted from December 2023 to April 2024. During the recruitment phase, participants completed an online questionnaire covering their height, weight, lifestyle, health status, diet, allergies, medication, and food supplement usage. Based on responses, the organizers selected 65 volunteers to participate in the intervention study, divided into three groups: control (CTRL), constipation (CONS), and antibiotic users (AB). The CTRL group comprised normal individuals with a body mass index (BMI) in the recommended range (18.5–25.0) and no reported health issues and medication usage. Participants with bowel movements occurring 2–3 times a week or less characterized by solid stools were assigned to the CONS group. The AB group included individuals who had used antibiotics within the past 6 months. The exclusion criteria for this study included severe or chronic diseases (e.g., cancer, Crohn's disease, ulcerative colitis), specific diets (like ketogenic, vegan, low carbohydrate high-fat diets, etc.), pregnancy or breastfeeding, frequent use of medications including non-prescription medications, a BMI below 18.5 (underweight), age under 18 years, and regular use of prebiotics or probiotics as food supplements. Participants were instructed to maintain their usual eating habits and lifestyle throughout the study. Those who did not comply with the study protocol were excluded from participation. Out of the 65 selected volunteers who were chosen for the study, 55 completed the study, of which 10 (18.2%) were men.

**Figure 1:**
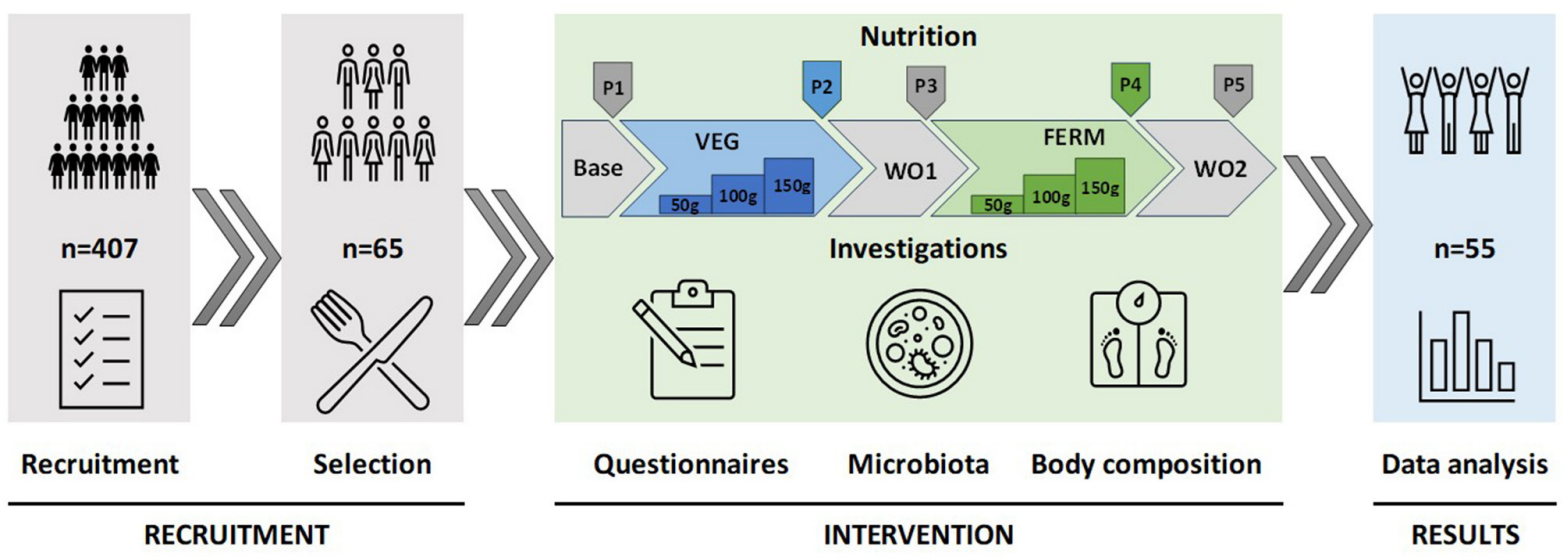
Design of the study. The study commenced with the recruitment and selection of participants, proceeded with an intervention phase involving the consumption of fresh and fermented vegetables in defined quantities over specified periods, and concluded with the analysis of data derived from questionnaires, gut microbiota sequencing, and body composition measurements. P1–P5 indicates time points for data collections. Base—base period (P1), regular eating habits and lifestyle, VEG—vegetable consumption period 3 weeks (P2), FERM—fermented vegetable consumption period 3 weeks (P4), WO1 (P3) and WO2 (P5)—washout periods, regular eating habits and lifestyle, 2 weeks each.

The intervention study consisted of five phases (Figure 1): a one-week base period, followed by two three-week test periods (VEG and FERM), separated by two-week washout periods (WO1 and WO2). The base period describes the starting point of the study according to participants regular eating habits and lifestyle. During the test periods, participants consumed fresh (VEG) or fermented (FERM) vegetables, one type per period, in addition to their regular menu. The VEG period included the consumption of additional fresh vegetables like carrot, kohlrabi and carrot-cabbage mixture. The FERM period included the consumption of FVs such as fermented carrot, fermented kohlrabi and kimchi. Vegetable intake during these periods increased gradually: 50 grams per day in the first week, 100 grams per day in the second week, and 150 grams of vegetables per day in the third week for both interventions. This incremental approach was designed to minimize potential side effects associated with increased fiber and fermented food intake. Two test periods were alternated with washout periods (WO1 and WO2) in purpose to restabilising the microbiota changes, caused by intervention. WO2 timepoint also indicates to the end of the study as final timepoint (P5). Both washout periods were 2 weeks long and participants were asked to follow their regular eating habits and lifestyle. The study followed a sequential intervention design without randomization; thus, the entire study cohort underwent the same order of vegetable consumption.

All participants signed written informed consent forms before the beginning of the study. The research was approved by the local ethics committee (Research Ethics Committee of the National Institute for Health Development, Reference number 1259, issued on 11/27/2023).

### 2.4 Data collection

At the end of each study period, fecal samples were collected (five fecal samples per participant) and body composition was measured (P1-P5). All fecal samples were collected by participants and transported to TFTAK within 24 hours after sampling. Body compositions were measured at TFTAK by instructed employee after each period. Different questionnaires, including microbiota samples (Supplementary Table 1) and frequency questionnaires about eating habits (Supplementary Table 2), were filled out online at the end of a specific period.

### 2.5 Physical examination

Participants' body composition was assessed using a Tanita body composition analyser (DC-360S, Tanita Corporation, Tokyo, Japan). In addition to weight in kilograms (kg) and body mass index (BMI), the analyser provided measurements for actual fat mass in kilograms, body fat percentage (FATP), and phase angle (PhA). Following Tanita's recommendations, a standardized bioelectrical impedance analysis (BIA) protocol was employed to ensure the most accurate results. Waist circumference (WC) was measured at the midpoint between the iliac crest and the bottom of the 12th rib, at the end of a normal expiration, and was recorded in centimeters (cm). The waist-to-height ratio (WHtR) was calculated by dividing waist circumference by height, with both measurements taken in the same units.

### 2.6 Questionnaires

At the end of each period, several questionnaires were completed. All the surveys, including the recruitment questionnaire, were conducted using RedJade sensory software version 6.1.0 (RedJade Sensory Solutions LLC, Martinez, CA, USA). A frequency questionnaire about participants' eating habits and choices was filled out after the 1st and 5th periods. Based on food frequency questionnaires, the consumption of major food groups was categorized as: most days, 2–5 days a week, once a week or less, and not consumed (Supplementary Table 2). The microbiota sample questionnaire was filled out at the end of each period. It collected detailed information on fecal sample consistency, the occurrence of side effects (e.g., bloating, flatulence), incidences of illness such as the common cold during the period, and the use of any medications (Supplementary Table 1).

### 2.7 Fecal sample collection, DNA extraction, and sequencing

Fecal samples were collected with DNA/RNA Shield Collection Tubes with Swabs (Zymo Research, Irvine, CA, USA) using FecesCatcher by TagHemi (Zeijen, The Netherlands) and stored at +4°C. Before DNA extraction, samples were frozen at −20°C at least overnight. DNA was extracted using the ZymoBIOMICS DNA Miniprep Kit (Zymo Research, Irvine, CA, USA) according to the manufacturer's instructions. Qubit™ 3 Fluorometer (Thermo Fisher Scientific, Waltham, MA, USA) and dsDNA BR Assay Kit (Thermo Fisher Scientific) were used for gDNA quantification.

The V4 hypervariable region of the 16S rRNA gene was PCR amplified using universal forward F515 5‘-GTGCCAGCMGCCGCGGTAA-3‘ and reverse R806 5'-GGACTACHVGGGTWTCTAAT-3' primers (23). Samples were sequenced using the Illumina MiSeq platform and 2 x 150 paired-end sequencing protocol. On average 30,988 reads (minimum 20 021 reads) per sample were obtained. The whole sequencing workflow was published before (24).

DNA sequence data was analyzed by BION-meta software (https://github.com/nielsl/mcdonald-et-al) according to the authors‘ instructions (25). The sequences were first cleaned at both ends using a 99.5% minimum quality threshold for at least 18 out of 20 bases for 5‘-end and 28 out of 30 bases for 3‘-end, followed by joining and removal of shorter contigs than 150 bp. Afterwards, the sequences were cleaned from chimeras and clustered by 95 % oligonucleotide similarity (k-mer length of 8 bp, step size 2 bp). Finally, consensus reads were aligned to the SILVA reference 16S rRNA database (version 138) using a word length of 8 and a similarity cut-off of 90 %.

### 2.8 Data grouping, clustering, and statistical analyses

**Figure 2:**
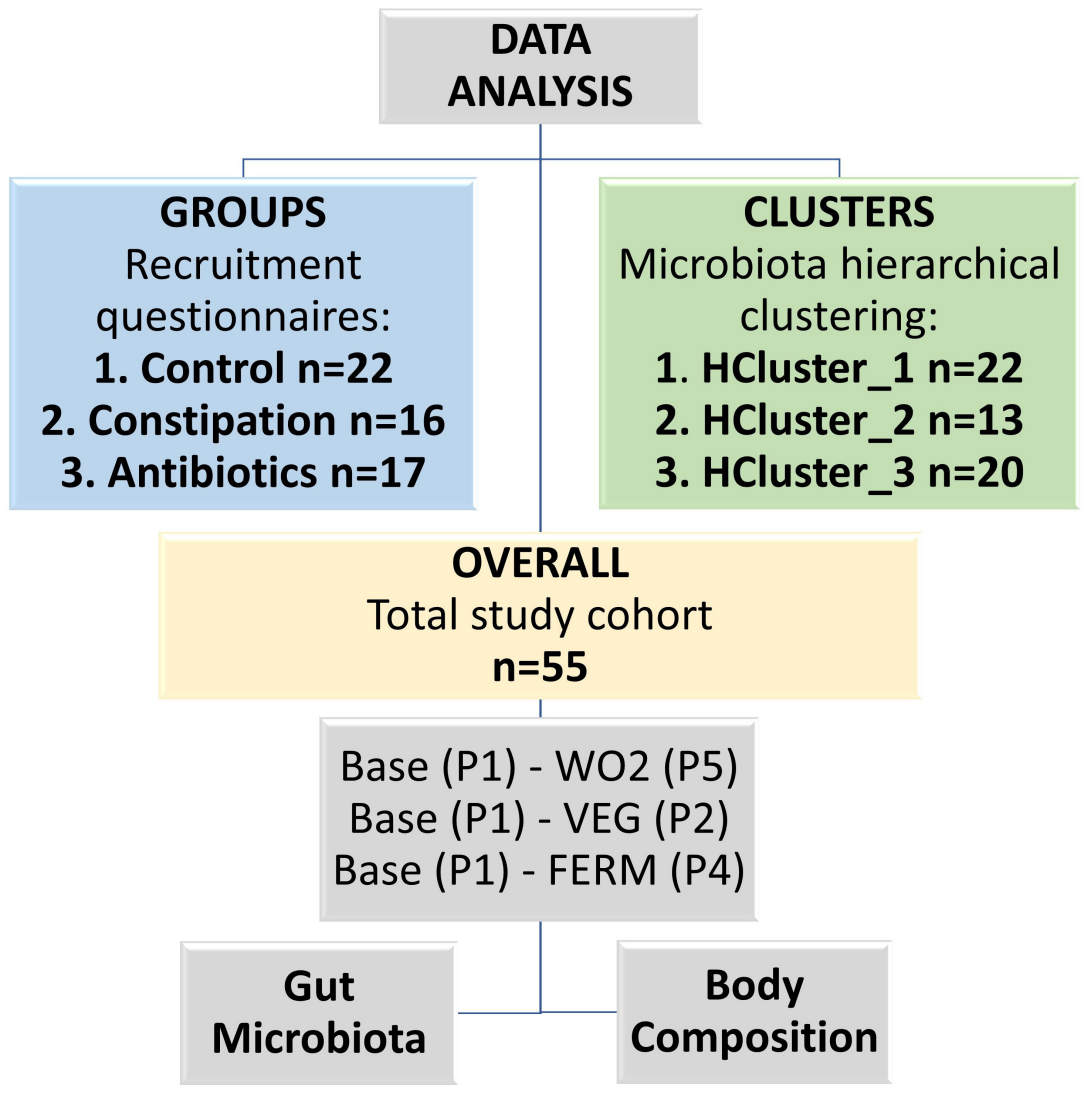
Scheme of the data analysis, which was conducted in three different branches (groups, clusters and overall study cohort). Base—baseline timepoint (P1), VEG—the end of the fresh vegetable consumption period (P2), FERM—the end of the fermented vegetable consumption period (P4), WO2—washout 2, the last timepoint of the study (P5).

Data analysis was performed in three branches (groups, clusters and overall study cohort) based on the results of the recruitment questionnaire (CTRL, CONS and AB groups), and microbiota taxonomical hierarchical clustering (HCluster_1-3) using Bray Curtis distance and Ward.D2 method. For both grouping approaches, gut microbiota and body composition were identified. Finally, similar analyses were performed for the whole study cohort. The Wilcoxon rank-sum test was used to evaluate differences in abundances of bacterial genera and body composition parameters between different study groups or clusters. The results of group and cluster comparisons, adjusted for multiple testing using the False Discovery Rate (FDR) method, are presented in the Supplementary Table 5. Pairwise comparisons were evaluated using Wilcoxon signed-rank test. All pairwise comparisons were calculated between the baseline (P1) and the end of the vegetable consumption (VEG, P2 or FERM, P4) or washout 2 (WO2, P5) periods (Figure 2).

Statistical analyses were performed at the bacteria genus level. Data analyses were done by R version 4.4.1 (The R Foundation for Statistical Computing, Vienna, Austria) using open public packages—vegan, cluster, pheatmap, ggpurb, Himsc, reshape2, tidyverse, patchwork and ggplot2 package were used for visualization.

## 3 Results

### 3.1 Microbial composition of fermented vegetable products consumed during the intervention study

**Figure 3:**
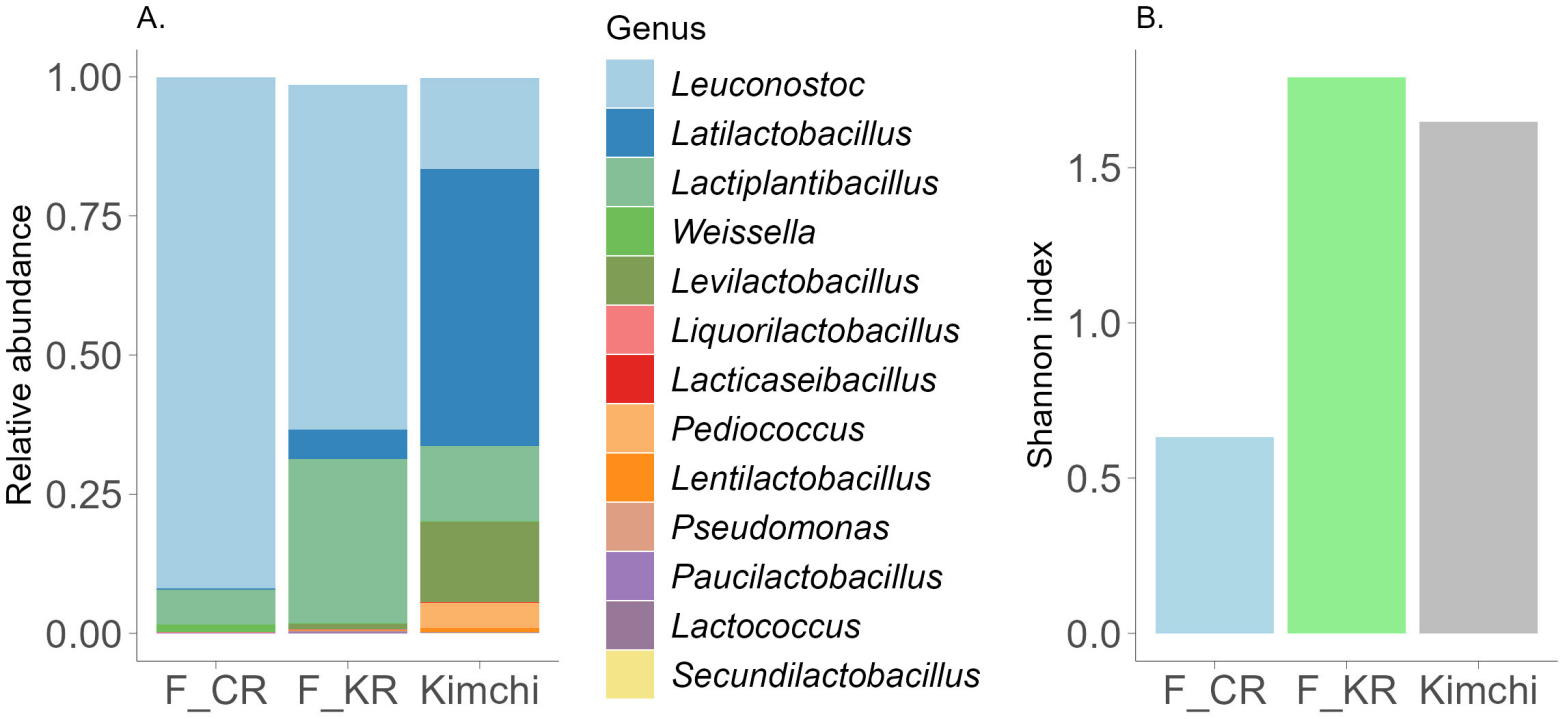
**(A)** Characterization of the fermented products microbiota composition at the genus level. Illustrated are genera with abundance >0.0005. F_CR—fermented carrot, F_KR—fermented kohlrabi. Y-axis—relative abundance normalized to 1, unclassified and genera lower than threshold (<0.0005) are not visualized. **(B)** Shannon index in different fermented products.

To understand the impact of everyday FV intake on gut microbiota, we had to characterize the microbial composition of consumed food, as fermented food-associated bacterial species may overlap with gut microbiota with definite consequences for human health. The microbiota composition of fermented carrot, kohlrabi and kimchi was defined, and the results are visualized in Figure 3A. All studied FVs had distinctive microbial signatures prevalent by lactic acid bacteria. Among all analyzed vegetables, fermented carrot microbiota diversity was the lowest with *Leuconostoc* dominant genus. The microbiota composition of fermented kohlrabi was similar to that of fermented carrots, although the proportions of *Latilactobacillus* and *Lactiplantibacillus* were higher in kohlrabi. Additionally, the Shannon index for kohlrabi was nearly three times higher than for carrots, indicating greater microbial diversity (Figure 3B). While kohlrabi exhibited the highest overall diversity based on the Shannon index, kimchi contained a higher level of diverse lactic acid bacteria, mainly consisting of *Latilactobacillus, Leuconostoc, Lactiplantibacillus* and *Levilactobacillus* (Figure 3).

### 3.2 Characterization of study groups (CTRL, CONS, AB) based on defined study criteria

#### 3.2.1 Baseline characterization of gut microbiota and body composition in defined study groups

**Figure 4:**
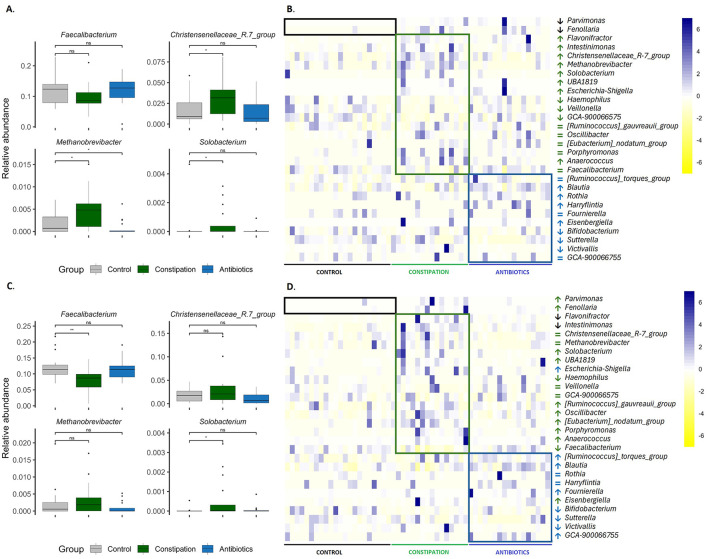
Differences in gut microbiota between groups **(A, B)** at the beginning of the study (P1) and **(C, D)** at the end of the study (P5) were assessed. **(A, C)** boxplots display the results of the Wilcoxon rank-sum test, with significance levels indicated as follows: *p < 0.05, **p < 0.01, and ns for statistically not significant. Heatmaps in **(B, D)** illustrate bacterial genera with varying abundances between groups, scaled by row. The boxes highlight bacterial genera that are differentially represented in the groups. Arrows indicate whether the abundance is statistically significantly higher (↑), lower (↓), or the same (=) compared to the control group. Unclassified data were removed, and the abundances were renormalized to 1. Black or gray indicates the control group (CTRL, *n* = 22), green represents the constipation group (CONS, *n* = 16), and blue signifies the antibiotics group (AB, *n* = 17).

**Figure 5:**
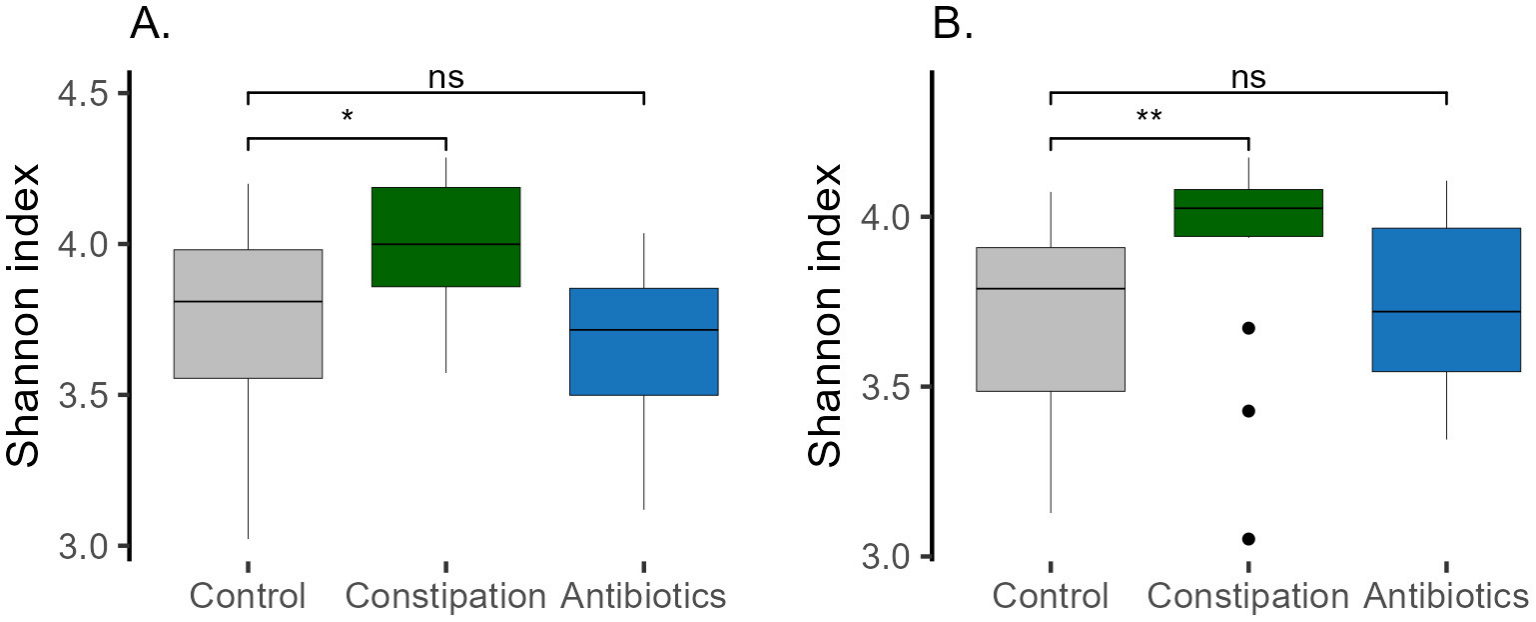
Shannon index in different groups **(A)** at baseline (P1), and **(B)** at the end of the study (P5, WO2). Wilcoxon rank-sum test was performed, *p < 0.05, **p < 0.01, ns, statistically not significant.

The main focus of the current study was to evaluate the impact of FV consumption on the gut microbiota of participants. We assumed that individuals with constipation problems and after a course of antibiotic treatment could benefit from FV consumption. The third CTRL group of volunteers who did not claim any health problems was taken as a comparison. Three target groups were formed based on the results of the self-reported recruitment questionnaire and their gut microbiota was identified before the intervention (Figure 2). All three studied groups had distinct microbial signatures at baseline (Figures 4A, B, Supplementary Figure 1A). Based on the Wilcoxon rank-sum test, the CTRL group had a lower abundance of the *Parvimonas* and *Fenollaria* genera than the other two groups. Members of the CONS group exhibited a higher proportion of the *Christensenellaceae R7* group*, Escherichia-Shigella, Methanobrevibacter, UBA1819, Intestinimonas, Flavonifractor, Solobacterium* and *Enterococcus* in contrast to the CTRL group. Additionally, decreased abundances of *GCA-900066575, Haemophilus*, and *Veillonella* were observed in the gut microbiota of the CONS group compared to that of the CTRL group. The AB group displayed a higher proportion of *Blautia* and *Eisenbergiella* and a lower proportion of *Bifidobacterium, Sutterella*, and *Victivallis*. Moreover, statistically significant differences were observed between the groups in bacterial genera with very low abundance levels, as detailed in Supplementary Figure 1A. Interestingly, the CONS group's Shannon index was statistically higher than the CTRL and AB groups (Figure 5), indicating a more diverse microbial composition.

Body composition was assessed using anthropometric measurements along with the results from a smart scale (Supplementary Figure 2A, Supplementary Table 3). Participants in the CONS and AB groups were significantly older than those in the CTRL group, with median ages of 40.5 and 40.0 vs. 35.5 years, respectively. The median body mass index (BMI) of participants was consistent across groups, maintaining a normal range of 23.6. However, compared to the CTRL group with a median fat mass percentage (FATP) of 23.6, a significantly higher FATP was observed in the CONS and AB groups, 29.8 and 27.1, respectively. Additionally, the CONS group exhibited a significantly higher median waist-to-height ratio (WHtR) than the CTRL group 0.50 vs. 0.46, respectively. Furthermore, the phase angle (PhA), which indicates cell metabolic health and membrane integrity, was lower in the AB group than in the CTRL group 5.6 vs. 6.0, respectively (Supplementary Figure 2A, Supplementary Table 3).

#### 3.2.2 Microbiota alterations in response to vegetable intake in study groups

**Figure 6:**
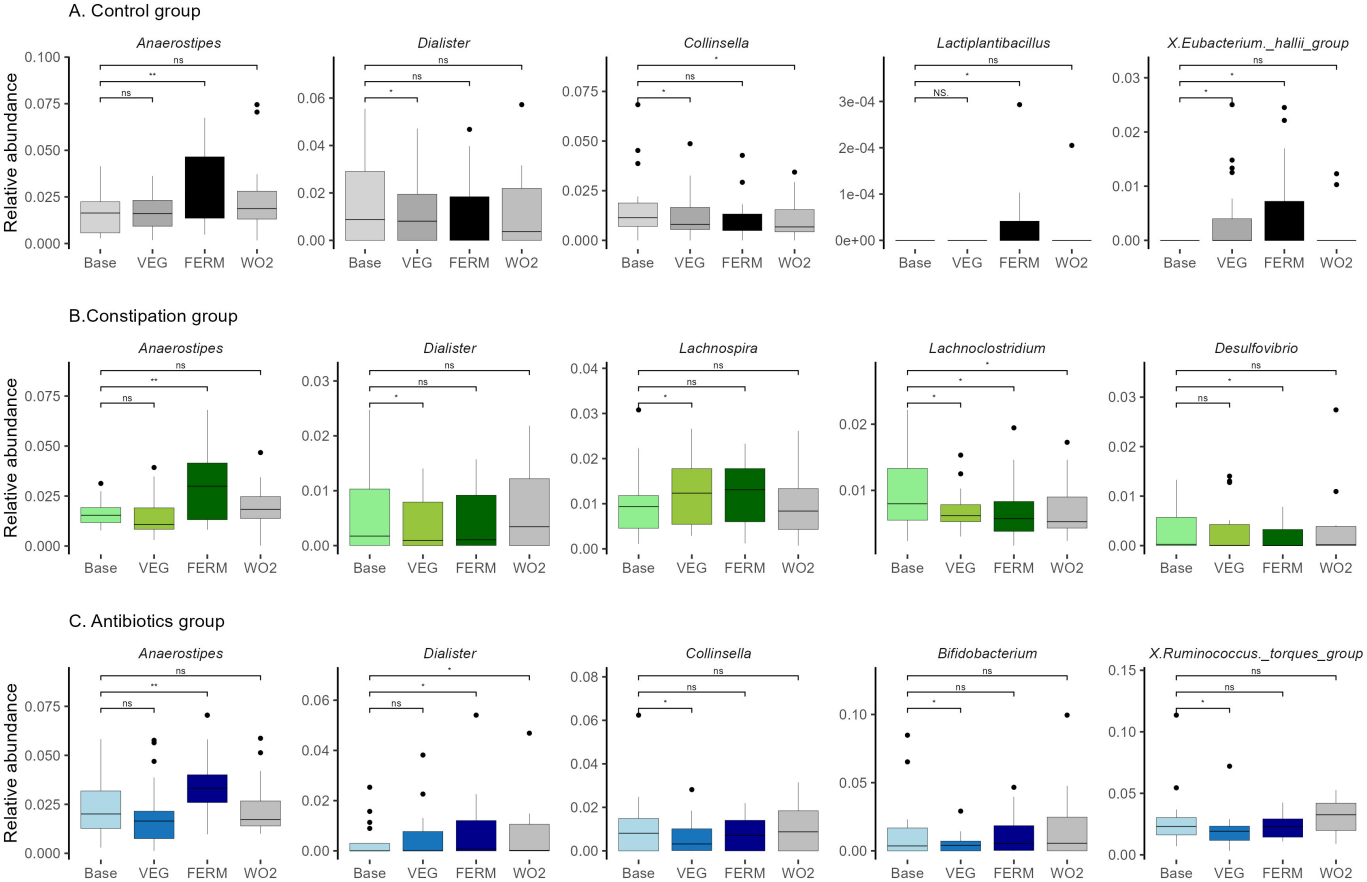
Relative abundance of bacterial genera in three groups: **(A)** control (CTRL, *n* = 22, gray), **(B)** constipation (CONS, *n* = 16, green), and **(C)** antibiotics (AB, *n* = 17, blue) group. Selected bacterial genera with statistically significant differences after the VEG (P2), FERM (P4) or WO2 (P5) period compared to the Base (P1) period sample are illustrated. Wilcoxon signed-rank test was performed, *p < 0.05, **p < 0.01, ns, statistically not significant. Unclassified taxa were removed and afterwards, abundances were renormalised to 1.

Next, we subsequently investigated the impact of increased consumption of fresh and fermented vegetables on the gut microbiota across three distinct groups. We observed the different effects of this intervention on gut microbiota composition between the studied groups (Figure 6, Supplementary Figure 3). In the CTRL group, *Anaerostipes* exhibited the most significant increase following the FERM period. Additionally, the abundances of *Butyricimonas, Lactiplantibacillus, Bacillus*, and *Pseudomonas* were elevated, while the *Lachnospiraceae NK4A136 group* showed a decrease after the FERM period. In contrast, the consumption of fresh vegetables led to a reduction in the relative abundances of *Collinsella, Dialister*, and *Coprobacter*, while increases were observed in the *Eubacterium nodatum* group and *Harryflintia*. Notably, the *Eubacterium hallii* group displayed a higher abundance following both the FERM and VEG interventions within the CTRL group's gut microbiota. Remarkably, the changes of the abundances of *Lachnospiraceae NK4A136 group, Bacillus* and *Collinsella* were detected even 2 weeks after the last intervention (WO2) (Figure 6A, Supplementary Figure 3A).

In the CONS group, results aligned with those observed in the CTRL group (Figure 6B, Supplementary Figure 3B), with *Anaerostipes, Bacillus*, and *Pseudomonas* showing increased abundances following FERM period, while *Dialister* exhibited a reduction after VEG period. Additionally, at the end of the FERM, the relative abundances of the *Eubacterium brachy group, Desulfovibrio, Parasutterella*, and *Hungatella* were notably reduced. Fresh vegetable consumption in the CONS group led to an elevation in *Lachnospira* and a reduction in the proportions of the *UCG-009* and *V9D2013 groups*. Both interventions resulted in decreased abundances of *Lachnoclostridium* and *UCG-008* in this group. Interestingly the vegetable effects on the abundances of *Bacillus, V9D2013_group* and *Lachnoclostridium* were still observed at the WO2 samples (Figure 6B, Supplementary Figure 3B).

In the AB group (Figure 6C, Supplementary Figure 3C), gut microbiota changes mirrored those observed in the other groups, with *Anaerostipes* and *Bacillus* showing increased abundances following FERM period. In contrast, *Dialister* exhibited an increase after the FERM period in this group, alongside increases in *Anaeroplasma* and *Acetobacter*. The VEG period negatively impacted the abundance of *Ruminococcus torques group, Bifidobacterium, Collinsella, Evtepia, Hungatella, Actinomyces*, and *Peptostreptococcus*. However, the *Eubacterium siraeum group* and *Coprobacter* increased following VEG term, while *Eisenbergiella* showed a slight reduction after both intervention periods in the AB group. Moreover, the vegetable effects on the abundances of *Dialiste*r, *Bacillus*, and *Coprobacter* were observed even at the WO2 timepoint (Figure 6C, Supplementary Figure 3C).

The abundances of *Anaerostipes* and *Bacillus* increased across all three groups following the consumption of FVs. Notably, *Bacillus* abundance remained elevated in the WO2 sample, whereas the effect on *Anaerostipes* was diminished following the washout period. In contrast, the genus *Dialister* exhibited varying responses among groups. Specifically, in the CTRL and CONS groups, the relative abundance of *Dialister* decreased after fresh vegetable intake. However, in the AB group, the abundance of *Dialister* increased following the FERM period and this effect persisted in the final sample (WO2) (Figure 6, Supplementary Figure 3).

Overall, the amount of Anaerostipes was significantly increased after the consumption of FVs for all studied groups. Hence, everyday increased vegetable and FV consumption impacts the gut microbiota.

#### 3.2.3 Comparative analysis of microbiota and body composition between study groups at the end of the study

At the end of the study, we conducted the same comparative analyses between groups as performed at the baseline, intending to determine whether increased vegetable and FV consumption had a beneficial impact on gut microbiota composition (Figures 4C, D, Supplementary Figure 1B). Interestingly, the microbiota of the CONST and AB groups exhibited certain similarities to that of the CTRL group. For instance, microorganisms associated with methane metabolism, such as the *Christensenellaceae R7 group* and *Methanobrevibacter*, no longer exhibited statistically significant differences in abundance between the CONS group and the CTRL group (Figure 4D, Supplementary Figure 1B). Furthermore, new differences in gut microbiota composition were observed between the groups by the end of the study. Both problematic groups, namely the CONS and AB groups, exhibited increased abundances of *Intestinimonas* and *Flavonifractor* compared to the CTRL group. Specifically, in the CONS group, *Ruminococcus gauvreauii group, Oscillibacter, Eubacterium nodatum group*, and *Porphyromonas* were present at significantly higher proportions (Figure 4D, Supplementary Figure 1B). In contrast, the abundance of *Faecalibacterium* was notably reduced in the CONS group (Figure 4C). In the AB group, elevated levels of *Ruminococcus torques group* and *Escherichia-Shigella* were detected compared to CTRL (Figure 4D, Supplementary Figure 1B).

At a lower abundance level, several notable changes in microbiota composition were observed. For instance, *Anaerococcus* remained at a higher relative abundance in the CONS group throughout the study. In the AB group, *Rothia* and *Harryflintia*, which exhibited elevated levels at the beginning of the study, showed a convergence toward levels observed in the CTRL group by the study's conclusion. Conversely, the proportion of *GCA-900066755* increased in the AB group at the end of the study (WO2). Detailed results are presented in Supplementary Figure 1B.

Similarly, the Shannon index of the CONS group remained the highest compared to the CTRL and AB groups, with its statistical significance even increasing by the end of the intervention (WO2) (Figure 5B).

In body composition analysis, the differences in PhA and WHtR between the groups disappeared by the end of WO2, indicating a positive impact of the consumed food on health indicators (Supplementary Figure 2B).

### 3.3 Characterization of clusters based on hierarchical clustering of participants' gut microbiota

#### 3.3.1 Baseline characterization of gut microbiota and body composition using a clustering approach

**Figure 7:**
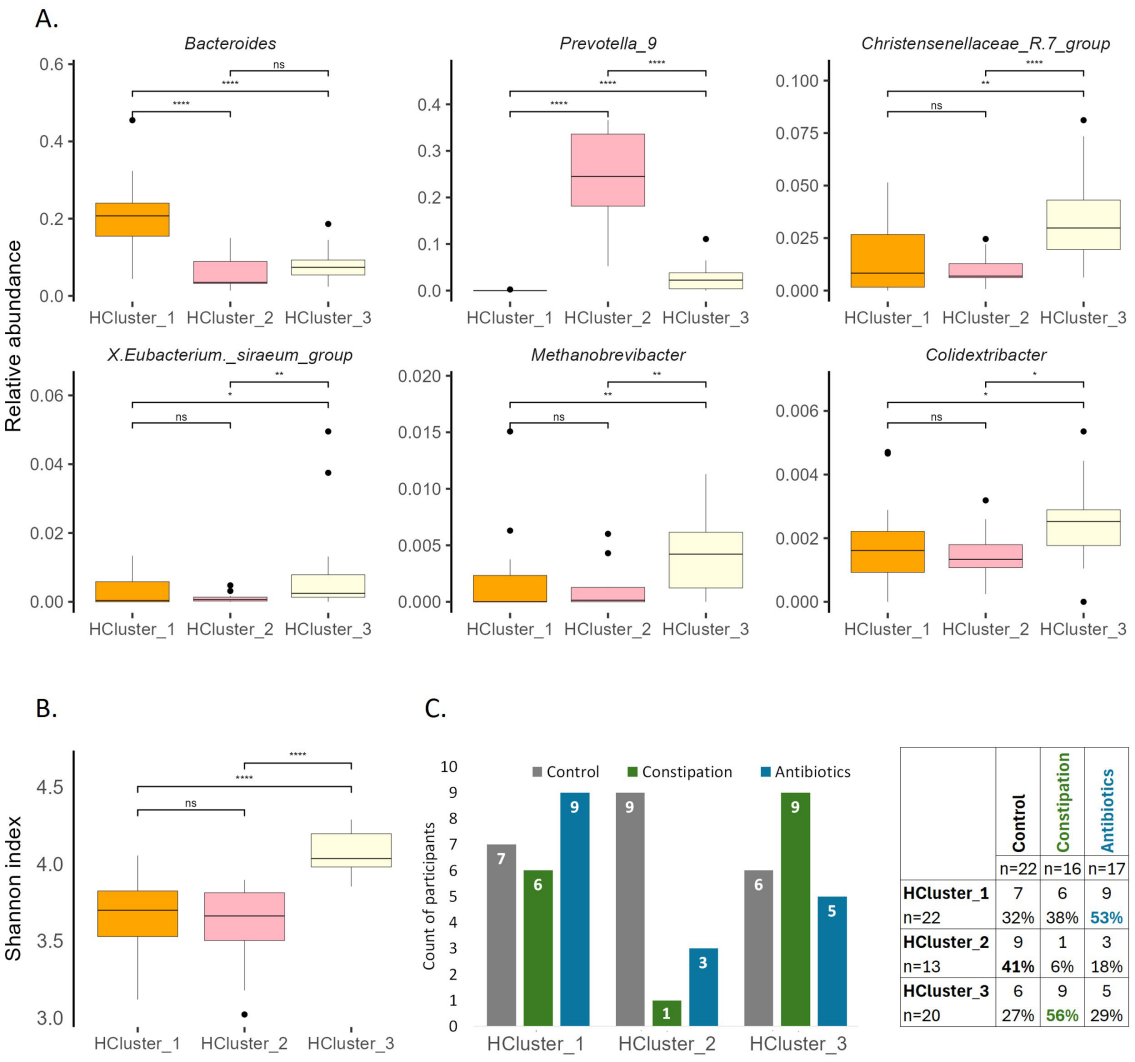
Characterization of clusters at baseline. **(A)** Selected bacterial genera illustrating the differences of gut microbiota between the clusters at baseline (P1). **(B)** Shannon index at baseline (P1). Wilcoxon rank-sum test was performed, *p < 0.05, **p < 0.01, ****p < 0.0001, ns, statistically not significant. **(C)** Division of participants between groups and clusters.

Next, we decided to group all study participants according to their microbiota types by the taxonomic hierarchical clustering method (Supplementary Table 1). The clustering analysis inside the current study shows three distinct microbiota types (Figure 7, Supplementary Figure 4), dominant by *Bacteroides* (HCluster _1), *Prevotella 9* (HCluster _2), and a more diverse group (HCluster _3) with increased abundances of *Christensenellaceae R7 group, X. Eubacterium siraeum group, Methanobrevibacter, Colidextribacter*, and a set of taxonomically unclassified and minor genres.

The Shannon index was the highest in HCluster_3 (Figure 7B) and remained the highest at the end of the WO2 (data not shown). This observation aligns with the alpha diversity results for the defined focus groups, where the CONS group demonstrated the highest Shannon index both at the beginning and end of the study (Figure 5).

The stratification analysis indicated that despite HCluster _1 being represented across all focus groups, with a slightly increased number of AB group members (53% of AB group participants belong to this cluster), HCluster _2 mainly consisted of the control cohort (41% of CTRL group participants belong to this cluster), and HCluster _3 dominated by CONS group participants (56% of CONS group participants belong to this cluster) (Figure 7C).

The median age of HCluster_3 was higher than that of HCluster_1 40 vs. 35 years, respectively. Regarding anthropometric data, the FATP of HCluster_3 was statistically higher than that of HCluster_2 26.9 vs. 21.0, respectively. This finding is logical, considering that the third cluster primarily comprised individuals dealing with constipation issues, which may be linked to their older age and higher fat percentage (Supplementary Figure 5A).

#### 3.3.2 Changes in gut microbiota in response to vegetable intake across clusters

**Figure 8:**
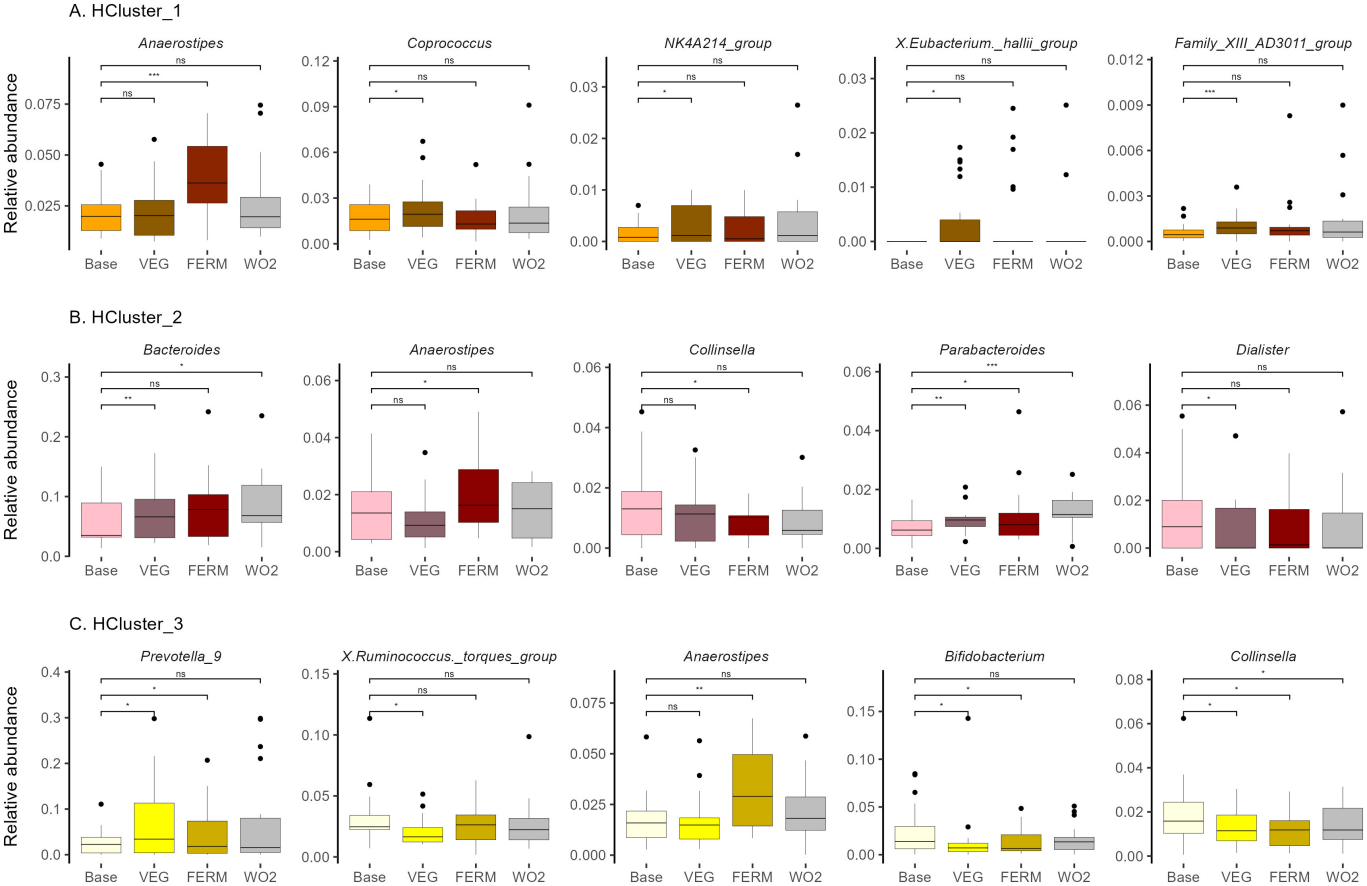
Relative abundance of bacterial genera in **(A)** HCluster_1 (*n* = 22), **(B)** HCluster_2 (*n* = 13), **(C)** HCluster_3 (*n* = 20). Bacterial genera with statistically significant differences after the VEG, FERM or WO2 period compared to the Base period sample are illustrated. Wilcoxon signed-rank test was performed, *p < 0.05, **p < 0.01, ***p < 0.001, ns –statistically not significant.

Subsequently, we investigated the response of the three clusters, identified based on gut microbiota hierarchical clustering to the intake of fresh and fermented vegetables (Figure 8, Supplementary Figure 6). HCluster_1 and HCluster_2 exhibited greater changes in response to fresh vegetable consumption than FV intake. In contrast, HCluster_3 demonstrated a more pronounced response to FV intake.

Across all clusters, an increase in the abundance of *Anaerostipes* was detected after FERM period, consistent with findings from group-based analyses (Figures 6, 8, Supplementary Figures 3, 6).

In HCluster_1, the genera *NK4A214 group* showed a significant increase following the VEG period (Figure 8A, Supplementary Figure 6A). Notably, the relative abundance of this genera, which was initially lower in this cluster compared to others at the beginning of the study (Supplementary Figure 4A), became comparable to that of the other clusters by the end of the study (data not shown). The relative abundance of the *Family XIII AD3011 group* increased after the VEG period (Supplementary Figure 6A) and was similar to HCluster_2 at the end of the study (Supplementary Figure 4B). Conversely, several butyrate-producing genera, including *Coprococcus* and *Eubacterium hallii group*, exhibited an increase following VEG period within this cluster, but these bacteria were not higher at the end of the study compared to other groups and the effects were not persistent (Figure 8A, Supplementary Figures 4B, 6A). In contrast, *UCG-003*, which exhibited an increase following both interventions, along with *Bacillus*, showed elevated levels after the FERM period, with the effects being sustained at the final time point (Supplementary Figure 6A).

In HCluster_2, the relative abundance of *Bacteroides* increased following the VEG period, while *Parabacteroides* exhibited an increase after all test periods. Both *Bacteroides* and *Parabacteroides* remained elevated at the final time point (WO2, P5). Notably the abundance level of *Bacteroides* remained significantly lower for HCluster_2 compared to HCLuster_1 at the end of the study (Supplementary Figure 4B). In contrast, the proportions of *Collinsella* and *Dialister* decreased following the FERM and VEG periods, respectively, but these effects were not persistent (Figure 8B). Additionally, a reduction in *Clostridium sensu stricto 1* and an increase in *Erysipelatoclostridium* were observed following the vegetable intake period. Notably, these microbial shifts persisted and remained stable at the final timepoint of the study (P5) (Supplementary Figure 6B).

In HCluster_3, the relative abundance of *Prevotella 9* increased following all test periods; however, this effect was transient. *Ruminococcus torques group* decreased after the intake of fresh vegetables, while *Bifidobacterium* abundance was reduced following the VEG and FERM periods, with these changes returning to baseline levels by the final time point. Conversely, *Collinsella* exhibited a decrease during both test periods and remained at a lower abundance at the end of the study within this cluster (Figure 8C). In HCluster_3, the genera *DTU089* and *V9D2013* showed a significant decrease following the VEG period (Supplementary Figure 6C). Notably, the relative abundance of these genera, which was initially higher in this cluster compared to others at the beginning of the study (Supplementary Figure 4A), became comparable to that of the other clusters by the end of the study (data not shown). Moreover, *Intestinibacter, Slackia, Candidatus Soleaferrea, UCG-008*, and *Bacillus* maintained the effects of vegetable intake at the study's conclusion (Supplementary Figure 6C).

#### 3.3.3 Differential microbial composition and body composition among clusters at study conclusion

The impact of the intervention varied among clusters. Across all clusters, the dominant genera remained consistently elevated throughout the study. In HCluster_1, at the end of the study, the levels of *Bacteroides, Holdemanella, Paraprevotella, Senegalimassilia, Howardella* and *Eggerthella* remained unchanged compared to the other clusters. The abundances of the *NKA214 group, Family XIII AD3011 group* and *UCG009* were lower at the beginning of the study compared to the other clusters. Conversely, *Fusicatenibacter* increased, while the abundances of *Victivallis* and *Pisum* were reduced in HCluster_1 at the end of the study compared to other clusters (Supplementary Figure 4B).

In HCluster_2, *Alistipes* and *UBA1819* were initially observed at lower levels than other clusters. By the end of the study, *Alistipes* had lower abundance only compared to HCluster_1, and *UBA1819* was similar in all clusters. In contrast, the proportion of *Subdoligranulum* and *Varibaculum* was significantly lower at the study's end than in other clusters. At the same time, *Prevotella 9* remained at a higher level, and *Merdibacter* stayed at a lower level at the WO2 time point relative to other clusters (Supplementary Figure 4B).

HCluster_3 exhibited the most variations. The abundances of five genera, including *UCG-002, Christensenellaceace R7 group, Methanobrevibacter, Family XIII AD3011 group* and *Pseudoflavonifractor*, remained stable across all interventions. Differences in *DTU89* and *V9D2013 group* disappeared. *Eubacterium siraeum group* was higher only compared to HCluster_1 at the end of the study. While *Colidextribacter, Oxalobacter* and *Peptostreptococcus* had elevated abundance compared to HCluster_2 in final timepoint. The abundance of *Gordonibacter* was lower than in other clusters. Meanwhile, compared to other cluster an increased proportion of *Defluviitaleaeceae UCG011* and *Porphyromonas* were observed in this cluster (Supplementary Figure 4B).

The median age of HCluster_3 stayed higher than that of HCluster_1. At the end of the study, significant statistical differences were observed between the first and third clusters regarding BMI and WHtR parameters, and between the second and third clusters concerning FATP (Supplementary Figure 4B).

### 3.4 Comparative analysis of grouping approaches for intervention responses in the Estonian gut microbiota cohort

**Figure 9:**
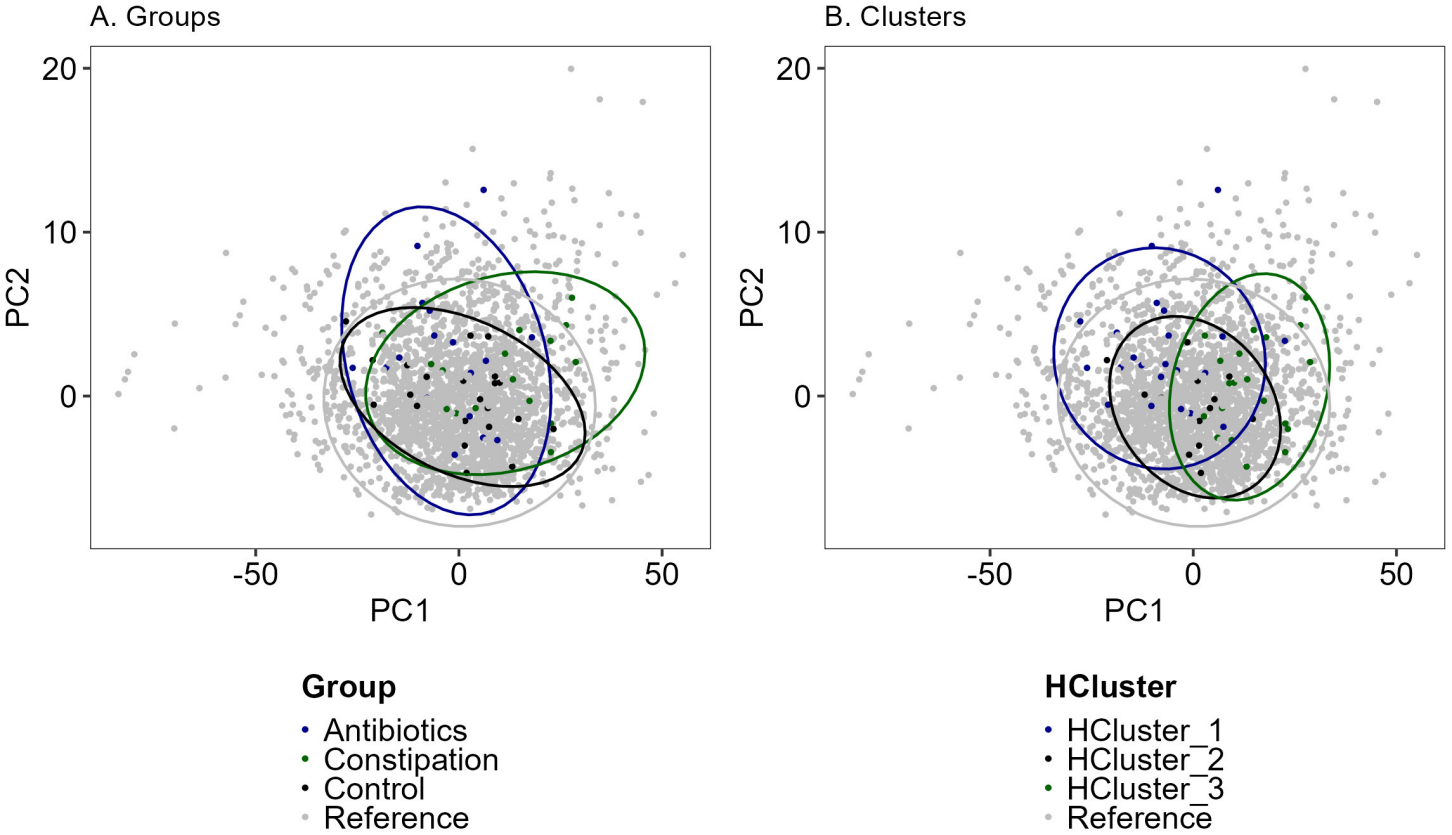
PCA (Principal component analysis) analysis of Estonian gut microbiota cohort (reference, *n* = 2,392, gray dots) and study cohort samples (*n* = 55). **(A)** In groups (black—control group, CTRL; green—constipation group, CONS; blue—antibiotics group, AB) and **(B)** in clusters (black—HCluster_2, green—HCluster_3, blue—HCluster_1). Unclassified genera were removed and thereafter renormalized to 1. CLR (Centered Log-Ratio Transformation) transformation was used before PCA calculations and scaled. Colors indicated different groups or clusters.

To better identify and position the global effects of the intervention, we conducted a comprehensive comparative analysis of both grouping methods within the Estonia gut microbiota cohort. The current data were mapped against the broader Estonian gut microbiota distribution dataset, comprising of 2,392 individuals (Figure 9). The two above-mentioned distinct grouping approaches based on self-reported health questionaries and taxonomic hierarchical clustering allowed for multiple comparative analyses and provided the validation for the initial grouping strategy. Subsequently, PCA cluster analysis was performed separately for the two formed groups.

Overall, the three identified groups and clusters closely resembled the dataset of the referenced Estonian gut microbiota cohort (Figure 9), particularly the control group and HCluster_2. Constipation and antibiotic groups showed slight deviations from the total cohort, but no significant separation in microbial composition was detected between these groups. Also, only a minor shift in samples from the constipation group and HCluster_3 was observed. The hierarchical clustering strategy provided the most consistent results throughout the intervention, highlighting the stability of this approach. Despite additional vegetable and FV consumption having minimal impact on overall gut microbiota composition, the antibiotic and constipation groups exhibited greater fluctuations across different intervention periods.

### 3.5 Overall response to vegetable intake in the entire study cohort

#### 3.5.1 Microbiota response to fresh and fermented vegetable intake in the entire study cohort

**Figure 10:**
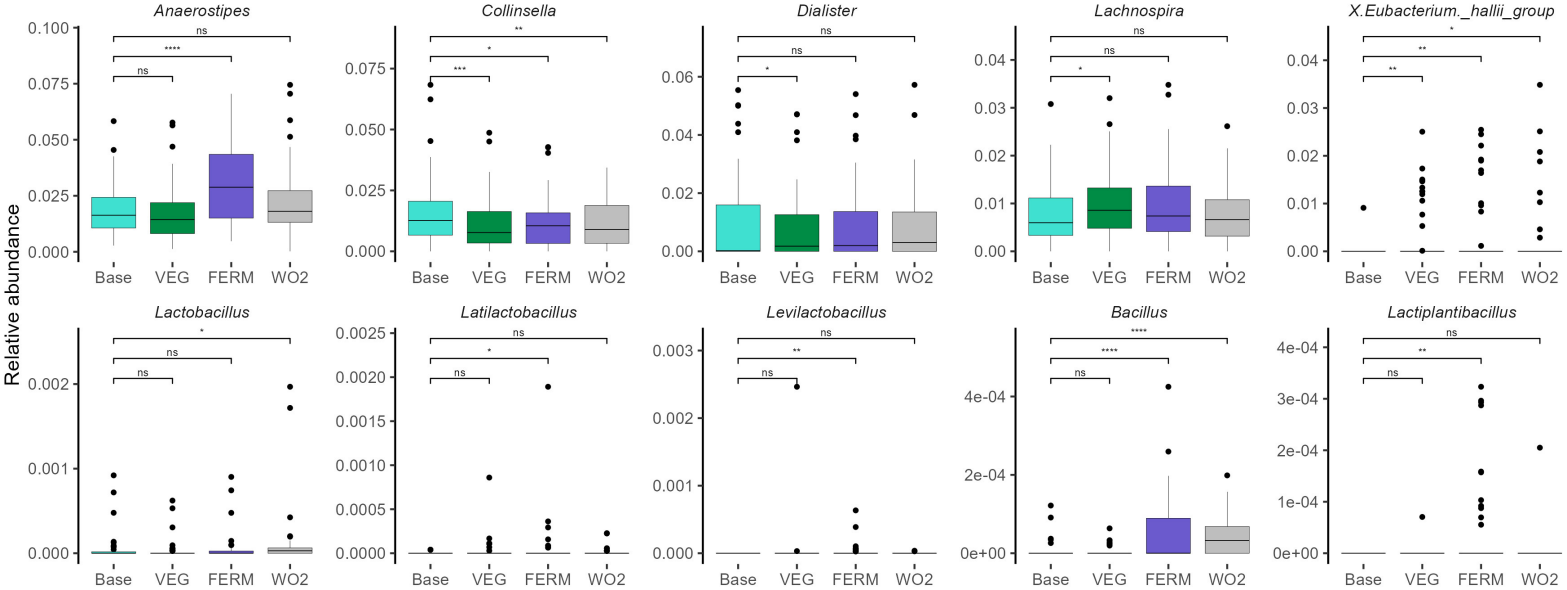
Selected bacterial genera illustrating the overall (*n* = 55) gut microbiota changes after different periods. Wilcoxon signed-rank test was performed, *p < 0.05, **p < 0.01, ***p < 0.001, ns, statistically not significant. Unclassified taxa were removed, and abundances were renormalised to 1.

Next, we applied a similar analytic strategy to the total study cohort (*n* = 55), identifying bacterial abundances across all intervention time points. We observed notable changes in gut microbiota composition following the intake of fresh and fermented vegetables (Figure 10, Supplementary Figure 7). The most pronounced shift in relative abundance was identified in the *Anaerostipes* genus after FERM period; however, this effect was transient and not evident at the end of the study. Additionally, an increase in *Lachnospira* and a decrease in *Dialister* were observed after the VEG period, and a decrease in *Negativibacillus* proportions after the FERM period, but these changes were not sustained.

Lactic acid bacteria, including *Latilactobacillus, Levilactobacillus*, and *Lactiplantibacillus*, originating from the FVs used in the study (Figure 3), exhibited statistically significant increases in relative abundance after the FERM period. However, these increases were not maintained at the final time point. In contrast, the reduction in *Collinsella* and the increase in the *Eubacterium hallii group*, noted after both vegetable intervention periods, remained statistically significant at the end of the study. Furthermore, the relative abundance of *Bacillus* showed a marked increase following the FERM period and remained elevated at the final timepoint (WO2). Abundance of *Lactobacillus* was increased only at the final timepoint sample (Figure 10, Supplementary Figure 7).

#### 3.5.2 Body composition changes after fresh and fermented vegetable intake in the entire study cohort

During the intervention study, changes in body composition were observed following the vegetable intake periods, with some of these alterations persisting beyond the study period.

The consumption of vegetables increased WC and WHtR, but there was no change in BMI. By the end of the study, both WC and WHtR decreased again. Additionally, PhA increased after both intervention periods, while a reduction in FATP was specifically observed after the fresh vegetable intake period. These changes were also noted at the end of the study (Supplementary Figure 8).

#### 3.5.3 Changes in nutritional habits following an intervention study

According to the frequency questionnaires, participants' daily consumption of fiber-rich and fermented foods was moderately low during the baseline period. On most days, no more than 50% of participants consumed fruits and vegetables. One-third regularly included whole grains in their diet, while only 2% consumed legumes. According to national dietary guidelines, these foods should be part of the daily menu (26). One third of participants consumed fermented dairy products daily, one fifth consumed nuts and seeds, while fewer than 5% ate fruits and vegetables (Supplementary Table 4). There was a change in the subjects' eating habits compared to the base period. Most importantly, the consumption of FVs changed significantly during the study. By the end of the WO2 period, 51% of participants reported consuming FVs at least twice a week (Wilcoxon signed-rank test, P= 0.001). Furthermore, 12.7% reported consuming them on most days compared to the baseline period. This level of consumption on most days was 3.5 times higher than during the baseline period when only 3.6% of participants had the same frequency of consumption. Even after the experimental period ended, the increased consumption continued to persist. Additionally, the participant's daily intake of sugary foods, especially added sugars, decreased. However, this change was not statistically significant (Supplementary Table 4). Otherwise, the participants' pre-study dietary habits remained largely unchanged, though there was a slight reduction in the intake of saturated fatty acid-rich products, such as sausages.

## 4 Discussion

Despite significant advances in gut microbiome research, the relationship between gut microbiota composition and its dynamic responses to dietary changes remains poorly understood, mainly due to the limited number of studies, variations in methodology based on different techniques and analytical pipelines, and participant characteristics. This underscores the need for further research in this area to draw robust conclusions and develop personalized dietary recommendations. Understanding the composition and variability of the gut microbiota in healthy or control groups remains the most important question to be addressed, as monitoring changes requires a solid baseline and variations in methodologies may lead to different conclusions.

It is increasingly recognized that consumption of fermented foods can influence the human gut microbiota, with potentially beneficial effects on a wide range of health parameters and conditions, including blood pressure, cholesterol levels, anxiety, depression, and skin conditions such as acne (27–30). While most available intervention studies have focused on yogurt and other dairy products, there remains a notable research gap regarding the effects of various fermented foods on the human gut microbiota (30). Therefore, this study aimed to evaluate the effects of FV consumption—specifically fermented carrots, fermented kohlrabi, and kimchi—on human health, with a particular emphasis on gut microbiota composition and function. In addition, we conducted a comprehensive analysis of the data using three distinct methodologies: grouping participants by pre-defined criteria, clustering samples by microbiota composition profiles, and considering the entire study cohort, which helped us to better interpret the data from different perspectives.

The results of different grouping and clustering approaches were quite similar. Thus, 53% of participants in the AB group were classified within HCluster 1, 41% of the CTRL group within HCluster 2, and 56% of the CONS group within HCluster 3, each with a specific microbiota pattern. This demonstrates some interoperability between methods but highlights the importance of grouping principles.

The most challenging outcome of the study, demonstrated through two different analytical approaches (grouping and clustering), showed that both the CONS group and members of HCluster 3 exhibited higher alpha diversity at baseline and at the final time point. Notably, 56% of participants from the CONS group belonged to this cluster. This phenomenon may be attributed to slower bowel transit, which provides an extended opportunity for bacteria to require more time for colonization and multiplication to establish themselves, in contrast to faster-growing species. Additionally, this group and cluster exhibited elevated levels of microorganisms associated with methane metabolism—*Christensenellaceae R7 group* and *Methanobrevibacter*. Methane production has been implicated in the slowing of fecal transit time, suggesting a mechanistic link between microbial composition, methane activity, and constipation (31). The convergence of these observations indicates a more profound interpretative significance of the results obtained.

Moreover, the CONS group and HCluster_3 exhibited an increased abundance of bacteria commonly associated with the oral microbiome, including *Fusobacterium, Porphyromonas*, and *Actinomyces*. Emerging evidence indicates that interactions between the oral and gut microbiomes may be more extensive than previously recognized (7). The potential transmission of these bacterial taxa has also been demonstrated by other studies (32). Importantly, several species within the genera displaying increased representation—such as *Fusobacterium, Solobacterium, Porphyromonas, Parvimonas*, and *Peptostreptococcus*—have been reported to be elevated in patients with colorectal cancer and are linked to choline metabolism (8, 33). These findings may provide critical insights into the interplay between gut microbiota composition, host health, and disease susceptibility, particularly in individuals experiencing constipation.

Regarding the significant health benefits of interventions, the study observed a reduction in bacterial taxa, including *Collinsella* and *Ruminococcus torques group*, after vegetable consumption in the AB group. These bacteria are associated with inflammatory processes, circulating insulin levels and mucin degradation, leading to decreased gut barrier function (34–36). To gain deeper insights into the impact of antibiotics on gut microbiota, it would be essential to monitor the specific treatment period, the type and dosage of antibiotics administered, and the timing between the completion of antibiotic therapy and microbiota sample collection. These factors are critical for accurately characterizing the effects of antibiotics on microbial composition and dynamics.

Nonetheless, a key objective in microbiome research is to identify microbial markers that are specific to particular diseases or health conditions. This often requires the comparison of different cohorts with appropriate control groups. In their recent publication, Joos et al. (9) proposed a roadmap for utilizing the gut microbiome as both a reporter and a predictor of health. The concept of a “healthy gut microbiota” is inherently complex and remains challenging to define (9, 37). Multiple factors must be considered, including individual variability, medication use, disease status, anthropometric parameters, and other variables, making identifying a universal standard for healthy gut microbiota challenging. Most individuals do not align perfectly with all these criteria. Despite extensive research efforts, the precise definition of a “healthy” gut microbiota remains ambiguous, creating challenges in interpreting the outcomes of nutritional intervention studies. While substantial knowledge exists regarding the roles of individual gut bacterial species in human health, the comprehensive analysis of microbiome data needs methodological adjustment. To address this, we applied multiple strategies for grouping participants according to their lifestyle or healthy status, enterotypes or analyzing the cohort as a whole. Additionally, we integrated the current study's findings with existing Estonian gut microbiota datasets to enhance the contextual understanding and interpretation of the intervention results. Comparison between the current study cohort and the Estonian population revealed subtle but more pronounced shifts in group or cluster characteristics than those observed due to the intervention. These findings offer valuable insights into the specific microbiota composition of the Estonian population, contributing to a more nuanced understanding of its unique features. In this study, we demonstrated that the gut microbiota profiles of the current study participants closely resemble those observed in the analyzed Estonian cohort (*n* = 2,392), regardless of their consumption of functional foods or the presence of health conditions. This finding highlights the substantial inter-individual and temporal variability inherent to human gut microbiota. The rationale behind this comparison was to assess the place of the normal control and focus groups within the Estonian cohort and to gain insight into the positioning of healthy gut microbiota. Previous research has shown that the closer an individual's microbiota is to the center, the healthier the gut microbiota (10). This branch theory that stresses not enterotypes but enterostates importance can be applied to interpret microbiota changes in dietary intervention studies, indicating the direction of positioning movement in response to food intake. Indeed, we observed that the CONS and AB groups were more dispersed and further from the central reference, paving the way for a deeper understanding of microbiota results and their interpretation. Moreover, the clusters identified in this study align with previously published data on the Estonian cohort, with *Bacteroides* and *Prevotella* emerging as the most distinguishable genera (38).

In recent years, there has been an increasing focus on analyzing microbiota data as compositional data, requiring specialized analytical approaches to ensure the accurate interpretation of microbial communities. This perspective emphasizes the need to account for the inherent relative nature of microbiota data, particularly to avoid overlooking bacterial taxa present at lower abundances. Such taxa, though less prominent, may play significant roles in the functionality and dynamics of the microbiome, necessitating methodologies that capture their contributions effectively (39, 40).

Finally, the results of our study were analyzed without applying any grouping or clustering methodologies, instead considering the entire study cohort as a single population. This approach facilitated the evaluation of the overall impact of increased consumption of fresh and fermented vegetables on human gut microbiota composition and associated health parameters. Overall, in the current intervention study, we observed a significant increase in the relative abundance of the *Anaerostipes* genus following FV consumption compared to the baseline for the whole cohort of participants. *Anaerostipes* is a beneficial commensal member of the gut microbiota, known for production of short-chain fatty acids (SCFAs), particularly butyrate. Through cross-feeding interactions with other bacteria, *Anaerostipes* can utilize acetate and lactate as substrates for butyrate synthesis, further supporting its role in promoting gut health (41). Furthermore, we observed an increase in lactic acid bacteria (LAB), particularly *Latilactobacillus, Levilactobacillus*, and *Lactiplantibacillus*, following the FERM period. Notably, these LAB genera were also identified in the microbiota of the FVs used in the study. This provides evidence supporting the role of fermented foods as a valuable source of viable beneficial bacteria, capable of transiently enhancing gut microbial composition (30, 34). In most cases, the effects of functional food consumption over a short duration are not sustained over time (42, 43). Similarly, in our study, the observed increases in LAB and *Anaerostipes* abundances diminished by the end of the study period. In contrast, the effects on *Eubacterium hallii group, Bacillus* and *Collinsella* were still evident at the final time point, 2 weeks after the cessation of FVs intake. Furthermore, the increased abundances of *Anaerostipes, Eubacterium hallii group*, and *Bacillus* may be linked to the possibly elevated lactate levels in the gut after the FERM period. This suggests that the enhanced lactate concentration in the digestive tract following FV consumption may support the growth of lactate-utilizing bacteria. Notably, both *Anaerostipes* and *Eubacterium hallii group* possess the capability to utilize lactate as a substrate for butyrate production (41, 44) with *Bacillus* being one of the producers of lactic acid (45). These observations highlight the variable response dynamics of different bacterial genera to dietary interventions, reflecting their distinct ecological roles and interactions within the gut microbiota.

The gut microbiota is pivotal in shaping human health across multiple dimensions. In the context of body composition, our study demonstrated that vegetable consumption may positively influence key body composition parameters. Two weeks after the FERM period, participants exhibited reductions in body FATP, compared to baseline measurements. Conversely, PhA showed significant increases. PhA, an indicator of cellular health, is particularly noteworthy; higher values are associated with greater cellularity, improved cell membrane integrity, and enhanced cell function (46). These findings suggest that dietary interventions, such as increased vegetable intake, support gut microbiota modulation and contribute to overall metabolic and cellular health improvements.

The study has certain limitations that warrant consideration. First, increasing the size of subgroups would enhance the precision of statistical analyses and improve the robustness of the results. Additionally, a higher participation rate among male subjects would also mitigate potential gender-related biases between groups, ensuring a more balanced dataset. Nonetheless, the study achieved a commendable completion rate, with 55 participants successfully finishing the extended intervention and a dropout rate of only 15%, reflecting the substantial commitment of the participants. In the main text, Wilcoxon rank-sum test results are presented without *p*-value adjustment, whereas FDR-adjusted *p*-values for group and cluster comparisons are reported in the Supplementary material. This approach was chosen to compare different analytical strategies and to avoid prematurely discarding potentially meaningful findings as false negatives due to multiple testing correction. It is acknowledged, however, that presenting unadjusted *p*-values may increase the risk of false positive results. Nevertheless, low-abundance taxa—often prone to loss of significance after adjustment—may play important roles in gut dysbiosis, and their exclusion could overlook biologically relevant signals.

Altogether, while the various grouping strategies revealed nuanced differences in the impact of fermented vegetable consumption on human health parameters, the overall beneficial influence of regular fermented food consumption on gut microbiota remains undoubtful. However, the idea behind group formation and the specific characteristics of the observed fluctuations plays a pivotal role in data interpretation. Furthermore, the selection and formation of target groups are critical to accurately assessing and understanding the true actual effects of the intervention.

## 5 Conclusions

This study provides a substantial contribution to the field by demonstrating that the effects of consuming fresh vs. fermented vegetables are highly dependent on the methodological framework used for grouping or clustering baseline samples. The findings underscore the complex interplay between dietary interventions, gut microbiota modulation, and health outcomes, emphasizing the pivotal role of analytical methodologies in shaping these interpretations. Our results highlight the significance of FVs as probiotic-potential dietary components, which can affect both the host's microbial composition and physiological parameters. Further investigations employing integrative and compositional analytical approaches are essential to advance our understanding of gut microbiota dynamics and their broader implications for human health.

## Data Availability

The original contributions presented in the study are publicly available. This data can be found here: https://www.ncbi.nlm.nih.gov, accession number PRJNA1216049.
