# Supplementary material for: Impact of fresh and fermented vegetable consumption on gut microbiota and body composition: insights from diverse data analysis approaches
Source: Front Nutr. 2025 Jul 15;12:1623710. doi: 10.3389/fnut.2025.1623710 (PMC12306187; doi:10.3389/fnut.2025.1623710)
Supplement: Supplementary file 1 [file Supplementary_file_1.zip › Supplementary Table 2.DOCX]

**STable 2:** Dietary habits questionnaire.

| **Food category** | | **Most days** | **2 to 5 days a week** | **Once a week or less** | **Not consumed** |
| --- | --- | --- | --- | --- | --- |
| **1** | **Vegetables and leafy greens** |  |  |  |  |
|  | **Fermented vegetables** |  |  |  |  |
|  | **Fruits and berries** |  |  |  |  |
|  | **Legumes** (peans, beans, etc.) |  |  |  |  |
| **2** | **Whole grain bread, rolls, pastries** |  |  |  |  |
|  | **Whole grain porrige, rice, pasta, muesli** |  |  |  |  |
|  | **Non-whole grain bread, rolls, pastries** |  |  |  |  |
|  | **Non-whole grain porrige, rice, pasta, muesli** |  |  |  |  |
|  | **Potatoes** |  |  |  |  |
| **3** | **Buttermilk, kefir, whey, sour cream, yogurts (plain), cheeses** |  |  |  |  |
|  | **Curd, cottage cheese** |  |  |  |  |
|  | **Milk and cream (10%, 35%)** |  |  |  |  |
|  | **Sweetened dairy products** |  |  |  |  |
| **4** | **Fish** |  |  |  |  |
|  | **Poultry** |  |  |  |  |
|  | **Red meat (beef, pork, lamb, game meat)** |  |  |  |  |
|  | **Sausages, frankfurters, wieners, semi-finished products, etc.** |  |  |  |  |
|  | **Egg** |  |  |  |  |
| **5** | **Seeds, nuts, almonds** |  |  |  |  |
|  | **Vegetable oils, avocado, olives** |  |  |  |  |
|  | **Butter, spreads, mayonnaise** |  |  |  |  |
| **6** | **Sugar, honey, jam, sweets, sweet and salty snacks** |  |  |  |  |
|  | **Soft drinks and juice beverages** |  |  |  |  |
| **7** | **Alcohol (strong alcohol, cocktails, beer, wine, cider, etc.)** |  |  |  |  |
