# Supplementary material for: Impact of fresh and fermented vegetable consumption on gut microbiota and body composition: insights from diverse data analysis approaches
Source: Front Nutr. 2025 Jul 15;12:1623710. doi: 10.3389/fnut.2025.1623710 (PMC12306187; doi:10.3389/fnut.2025.1623710)
Supplement: Supplementary file 1 [file Supplementary_file_1.zip › Supplementary Table 1.DOCX]

**STable 1**

**The microbiota sample questionnaire**

1. Have you had any cold-related illnesses (cough, runny nose, sore throat, etc.) and taken any medications (ibuprofen, paracetamol, aspirin) in the past week?
   1. No
   2. Yes, without fever and without medications
   3. Yes, without fever, but with the use of over-the-counter medications
   4. Yes, with fever and the use of over-the-counter medications
   5. Yes, with fever and/or the use of prescription medications
2. How would you describe the consistency of the specific fecal sample?
   1. Solid
   2. Intermediate
   3. Soft
3. Have you experienced any digestive problems in the past week?
   1. No
   2. Yes, constipation
   3. Yes, diarrhea
   4. Yes, flatulence/bloating
   5. Yes, other, please specify
4. Did you consume the specified amount of vegetables provided in the study? Please answer only after the period of consuming non-fermented/fermented vegetables has ended (for P2 and P4).
   1. Yes, as specified
   2. Deviated, less than specified, please explain
   3. Deviated, more than specified, please explain
5. How did you consume the vegetables specified in the study? Please answer only after the period of consuming non-fermented and fermented vegetables has ended (for P2 and P4).
   1. Raw
   2. Cooked
   3. Both ways
