# Supplementary material for: Impact of fresh and fermented vegetable consumption on gut microbiota and body composition: insights from diverse data analysis approaches
Source: Front Nutr. 2025 Jul 15;12:1623710. doi: 10.3389/fnut.2025.1623710 (PMC12306187; doi:10.3389/fnut.2025.1623710)
Supplement: Supplementary file 1 [file Supplementary_file_1.zip › Supplementary Material Captions.DOCX]

Supplementary Material

**Supplementary Figures and Tables:**

**SFig 1:** Gut microbiota differences of the groups at genus level. **A.** at baseline (P1) and **B.** at the end of the study (P5). Wilcoxon rank-sum test was performed, **p*<0.05, ***p*<0.01, ****p*<0.001, ns - statistically not significant. Unclassified taxa were removed and afterwards, abundances were normalised to 1.

**SFig 2:** Body composition differences of the groups. **A.** at baseline (P1) and **B.** at the end of the study (P5). Wilcoxon rank-sum test was performed, **p*<0.05, ***p*<0.01, ns - statistically not significant. BMI- Body mass index, WC – waist circumference in cm, FATP – body fat percent, PhA – phase angle, WHtR – waist to height ratio.

**SFig 3:** Relative abundance of bacterial genera in three groups: **A.** control (n=22, grey), **B.** constipation (n=16, green), and **C.** antibiotics (n=17, blue) group. Bacterial genera with statistically significant differences after the VEG (P2), FERM (P4) or WO2 (P5) period compared to the Base (P1) period sample are illustrated. Wilcoxon signed-rank test was performed, **p*<0.05, ***p*<0.01, ns – statistically not significant. Unclassified taxa were removed and afterwards, abundances were renormalised to 1.

**SFig 4:** Gut microbiota differences of the clusters at genus level. **A.** at baseline (P1) and **B.** at the end of the study (P5). Wilcoxon rank-sum test was performed, **p*<0.05, ***p*<0.01, ****p*<0.001, ns - statistically not significant. Unclassified taxa were removed and afterwards, abundances were normalised to 1.

**SFig 5:** Body composition differences of the clusters. **A.** at baseline (P1) and **B.** at the end of the study (P5). Wilcoxon rank-sum test was performed, **p*<0.05, ***p*<0.01, ns - statistically not significant. BMI- Body mass index, WC – waist circumference in cm, FATP – body fat percent, PhA – phase angle, WHtR – waist to height ratio.

**SFig 6:** Relative abundance of bacterial genera in three clusters: **A.** HCluster_1 (n=22, orange), **B.** HCluster_2 (n=13, pink), and **C.** HCluster_3 (n=20, yellow). Bacterial genera with statistically significant differences after the VEG (P2), FERM (P4) or WO2 (P5) period compared to the Base (P1) period sample are illustrated. Wilcoxon signed-rank test was performed, **p*<0.05, ***p*<0.01, ****p*<0.001, ns – statistically not significant. Unclassified taxa were removed and afterwards, abundances were renormalised to 1.

**SFig 7:** Relative abundance of bacterial genera in overall study cohort (n=55). Bacterial genera with statistically significant differences after the VEG (P2, green), FERM (P4, purple) or WO2 (P5, grey) period compared to the Base (P1, turquoise) period sample are illustrated. Wilcoxon signed-rank test was performed, **p*<0.05, ***p*<0.01, ****p*<0.001, ns – statistically not significant. Unclassified taxa were removed and afterwards, abundances were renormalised to 1.

**SFig 8:** Changes in body composition in all study participants (n=55) after fresh vegetable (P2, VEG), fermented vegetable (P4, FERM) intake and at the end of the study (P5, WO2) compared to base period sample (P1). Wilcoxon signed-rank test was performed, **p*<0.05, ***p*<0.01, ****p*<0.001, ns – statistically not significant. BMI- Body mass index, WC – waist circumference in cm, FATP – body fat percent, PhA – phase angle, WHtR – waist to height ratio.

**STable 1:** The microbiota sample questionnaire.

**STable 2**: Frequency questionnaire about dietary habits. Foods are divided into 7 main categories and frequencies are grouped as - most days, 2 to 5 days a week, once a week or less or not consumed.

**STable 3: A.** Participant group and cluster assignments, along with baseline body composition results. **B.** The table presents the average and mean values of body composition parameters within groups, clusters, and overall, accompanied by standard deviations and quartiles.

**STable 4:** Consumption of fiber-rich, fermented and sugary foods. Significant change is marked with *, Wilcoxon signed-rank test was performed, *p* = 0.01. ≥ 2 days/week – the sum of answers most days and 2 to 5 days a week, ≤ 1 day/ week or less – once a week or less, never – not consumed.

**STable 5:** FDR-adjusted results of group and cluster comparisons following Wilcoxon rank-sum tests are reported at baseline (P1) and at the end of the study (P5). Only statistically significant adjusted *p*-values are presented. For cluster comparisons, only genera that differ significantly from at least two other clusters are included. **p*<0.05, ***p*<0.01, ****p*<0.001, *****p*<0.0001. n1, n2 – number of participants in group or cluster.
