# Supplementary material for: Impact of fresh and fermented vegetable consumption on gut microbiota and body composition: insights from diverse data analysis approaches
Source: Front Nutr. 2025 Jul 15;12:1623710. doi: 10.3389/fnut.2025.1623710 (PMC12306187; doi:10.3389/fnut.2025.1623710)
Supplement: Supplementary file 1 [file Supplementary_file_1.zip › Supplementary Table 4.DOCX]

**STable 4:** Consumption of fiber-rich, fermented and sugary foods. Significant change is marked with *, p = 0.01, Wilcoxon signed-rank test was performe. ≥ 2 days/week – the sum of answers most days and 2 to 5 days a week, ≤ 1 day/ week or less – once a week or less, Never – not consumed.

| **Food group** | **Base period, n (%)** | **Washout 2 (WO2), n (%)** |
| --- | --- | --- |
| **Vegetables and leafy salads** |  |  |
| ≥ 2 days/week | 54 (98.2) | 53 (96.4) |
| ≤ 1 day/ week or less | 1 (1.8) | 2 (3.6) |
| Never | 0 | 0 |
| **Fermented vegetables*** |  |  |
| ≥ 2 days/week | 11 (20.0) | **28 (50.9)** |
| ≤ 1 day/ week or less | 39 (70.9) | 22 (40.0) |
| Never | 5 (9.1) | 5 (9.1) |
| **Legumes** |  |  |
| ≥ 2 days/week | 24 (43.6) | 20 (36.4) |
| ≤ 1 day/ week or less | 28 (50.9) | 33 (60.0) |
| Never | 3 (5.5) | 2 (3.6) |
| **Fruits and berries** |  |  |
| ≥ 2 days/week | 49 (89.1) | 48 (87.3) |
| ≤ 1 day/ week or less | 6 (10.9) | 7 (12.7) |
| Never | 0 | 0 |
| **Whole grain bread** |  |  |
| ≥ 2 days/week | 40 (72.7) | 40 (72.7) |
| ≤ 1 day/ week or less | 11 (20.0) | 13 (23.6) |
| Never | 4 (7.3) | 2 (3.6) |
| **Fermented dairy products** |  |  |
| ≥ 2 days/week | 38 (69.1) | **44 (80.0)** |
| ≤ 1 day/ week or less | 16 (29.1) | 10 (18.2) |
| Never | 1 (1.8) | 1 (1.8) |
| **Nuts and seeds** |  |  |
| ≥ 2 days/week | 36 (65.5) | 32 (58.2) |
| ≤ 1 day/ week or less | 18 (32.7) | 23 (41.8) |
| Never | 1 (1.8) | 0 |
| **Sugary foods** |  |  |
| ≥ 2 days/week | 46 (83.6) | 40 (72.7) |
| ≤ 1 day/ week or less | 7 (12.8) | 15 (27.3) |
| Never | 2 (3.6) | 0 |
