# Supplementary figures and images for: Impact of fresh and fermented vegetable consumption on gut microbiota and body composition: insights from diverse data analysis approaches
Source: Front Nutr. 2025 Jul 15;12:1623710. doi: 10.3389/fnut.2025.1623710 (PMC12306187; doi:10.3389/fnut.2025.1623710)

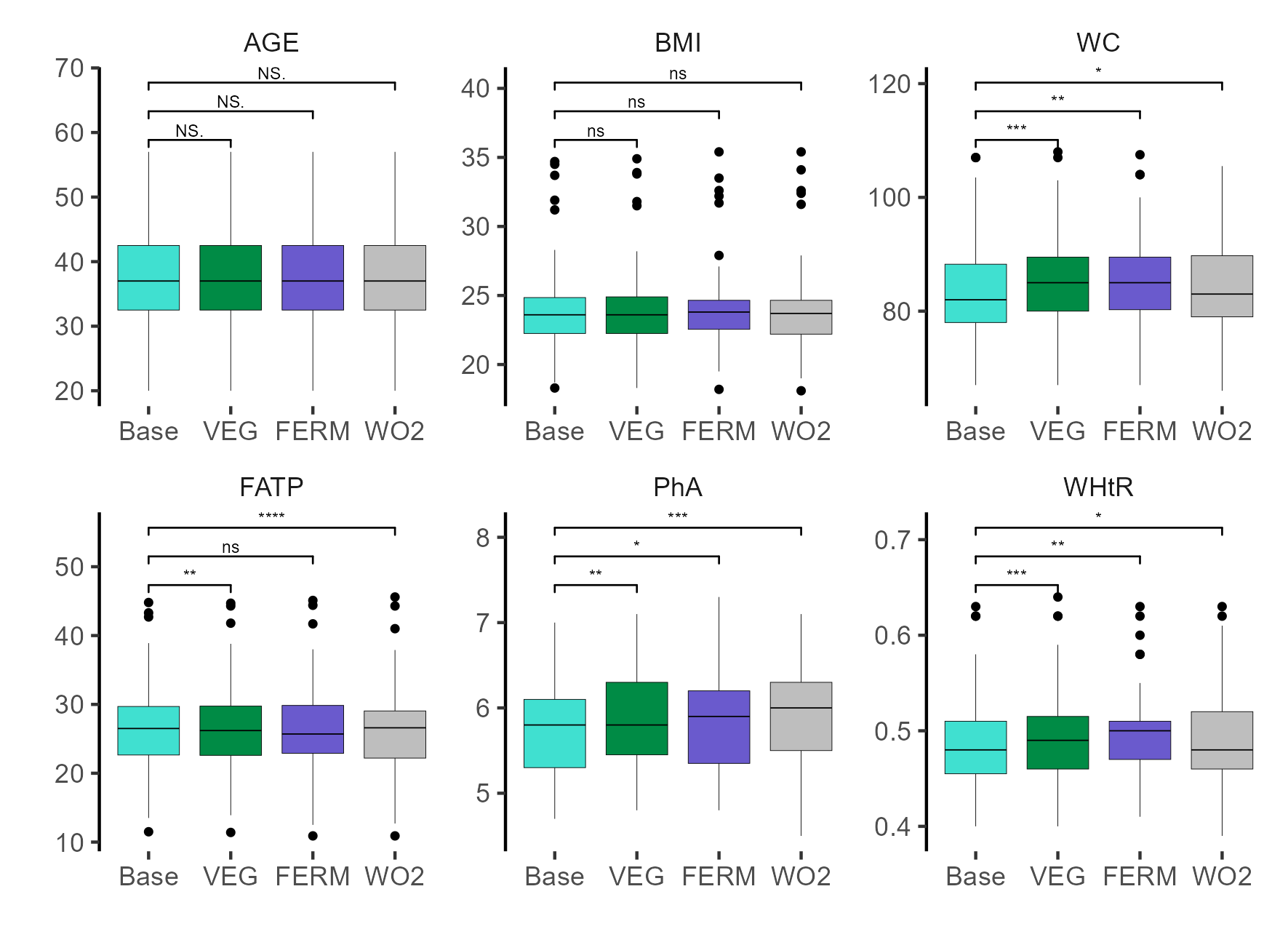

Supplement: Supplementary file 1 [file Supplementary_file_1.zip › Supplementary Figure 8.TIFF]

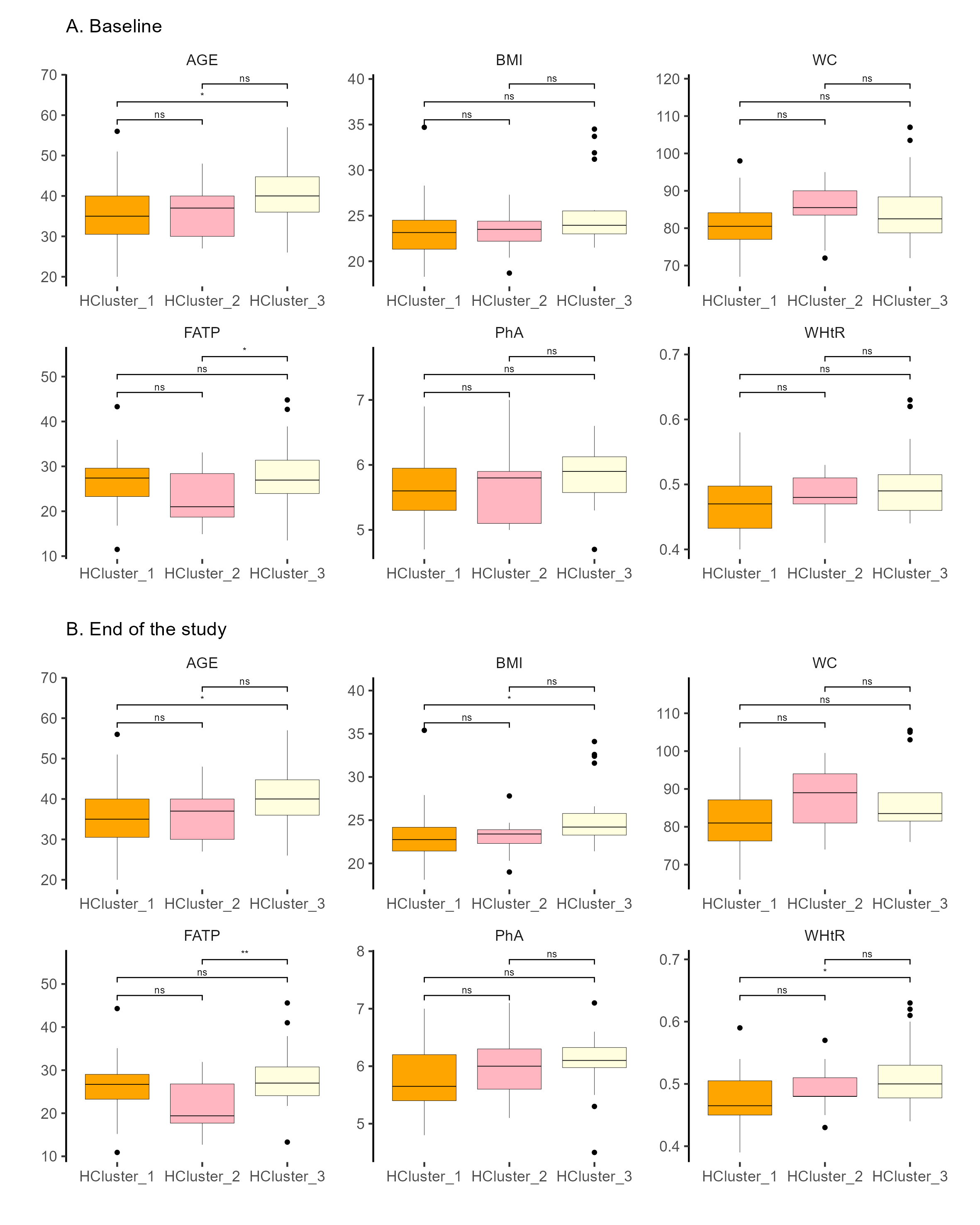

Supplement: Supplementary file 1 [file Supplementary_file_1.zip › Supplementary Figure 5.TIFF]

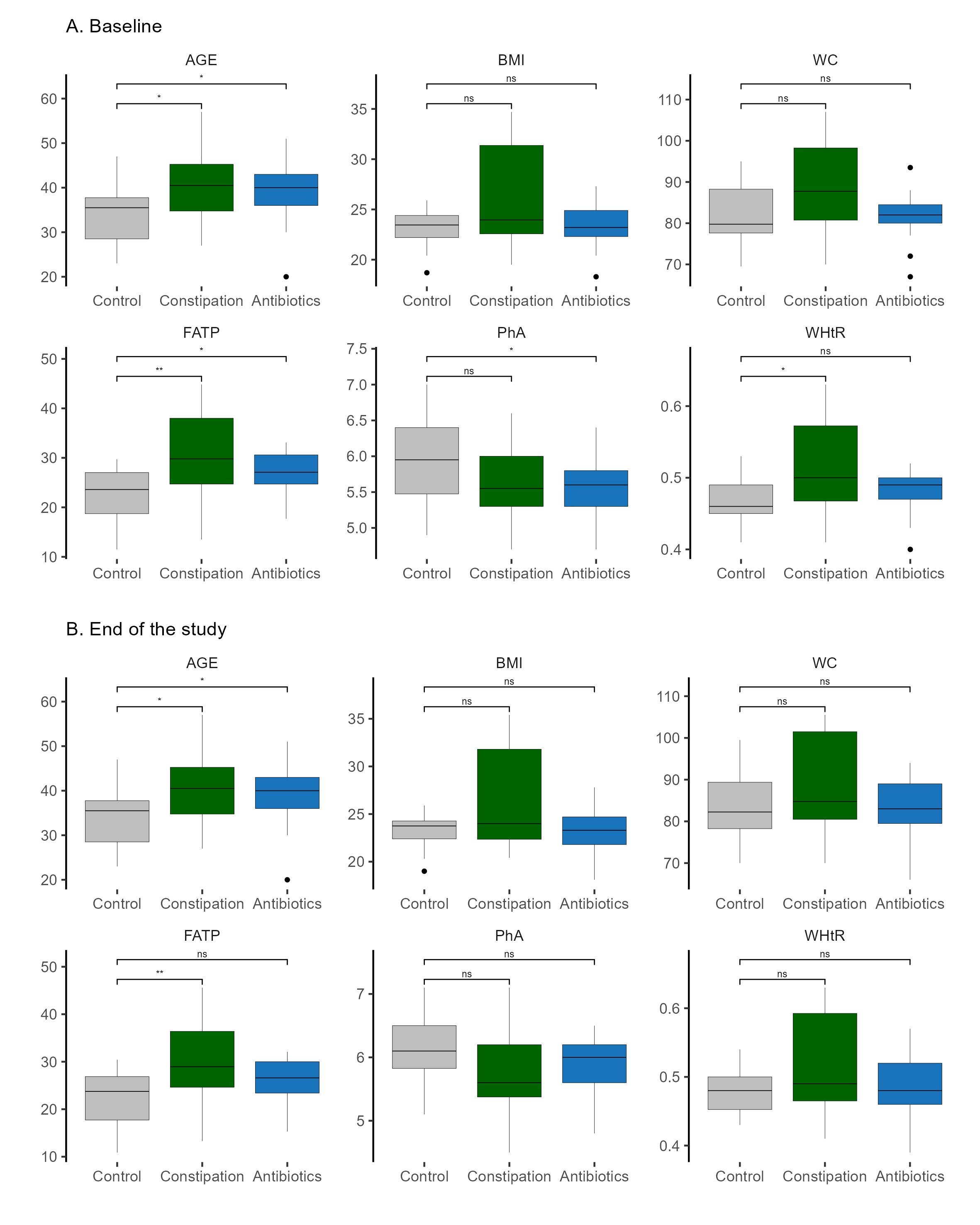

Supplement: Supplementary file 1 [file Supplementary_file_1.zip › Supplementary Figure 2.TIFF]

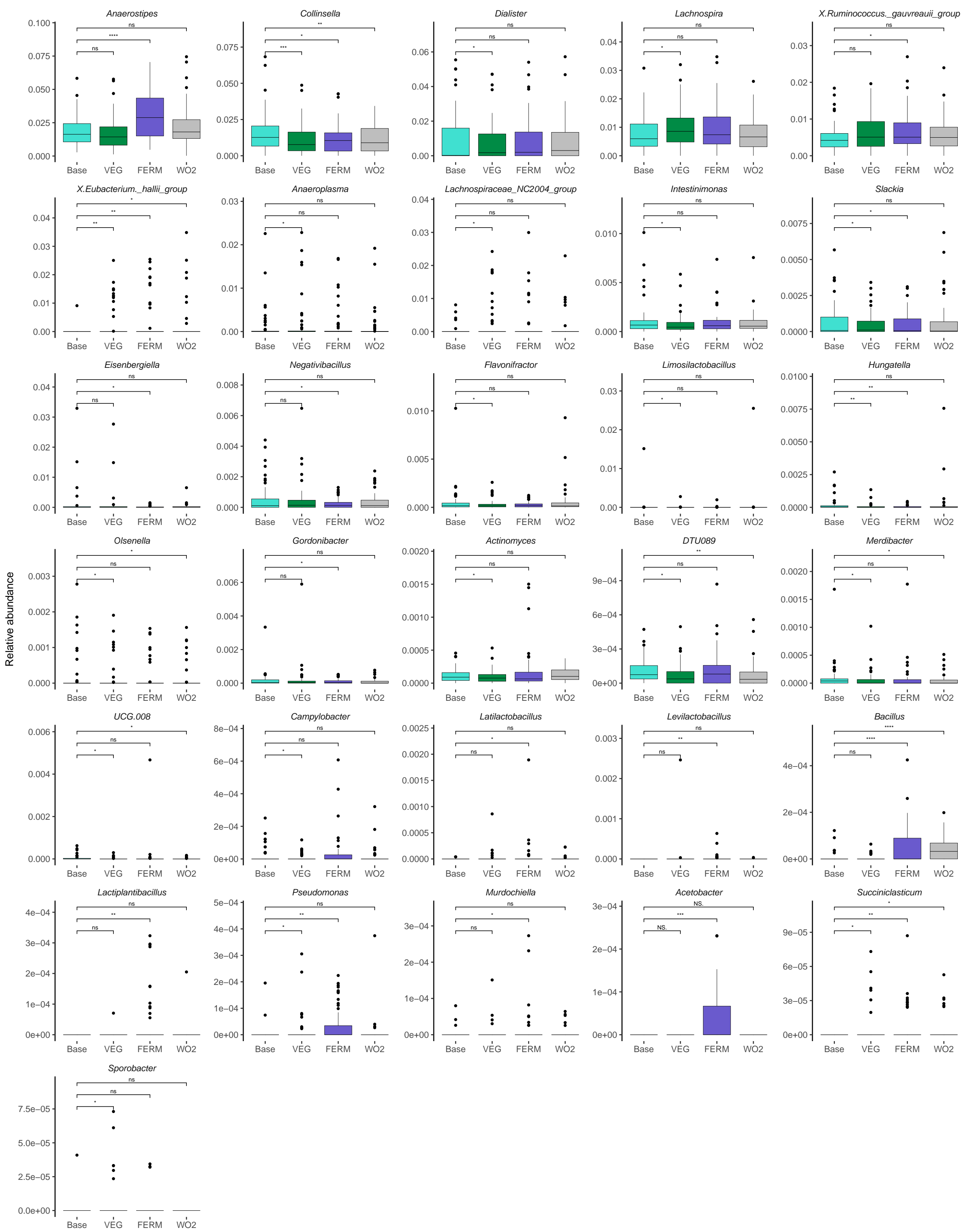

Supplement: Supplementary file 1 [file Supplementary_file_1.zip › Supplementary Figure 7.PDF]

A. HCluster\_1

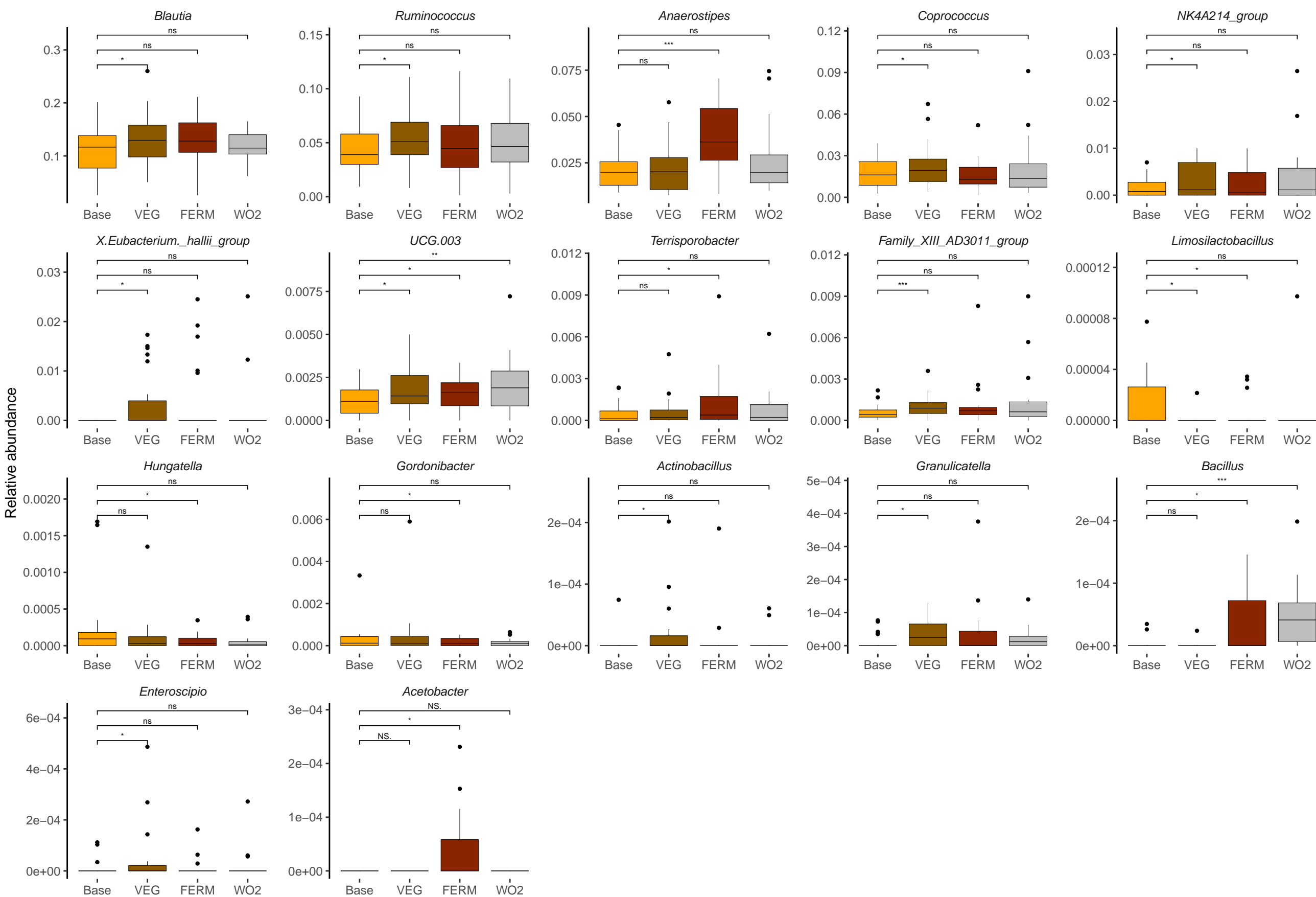

B. HCluster\_2

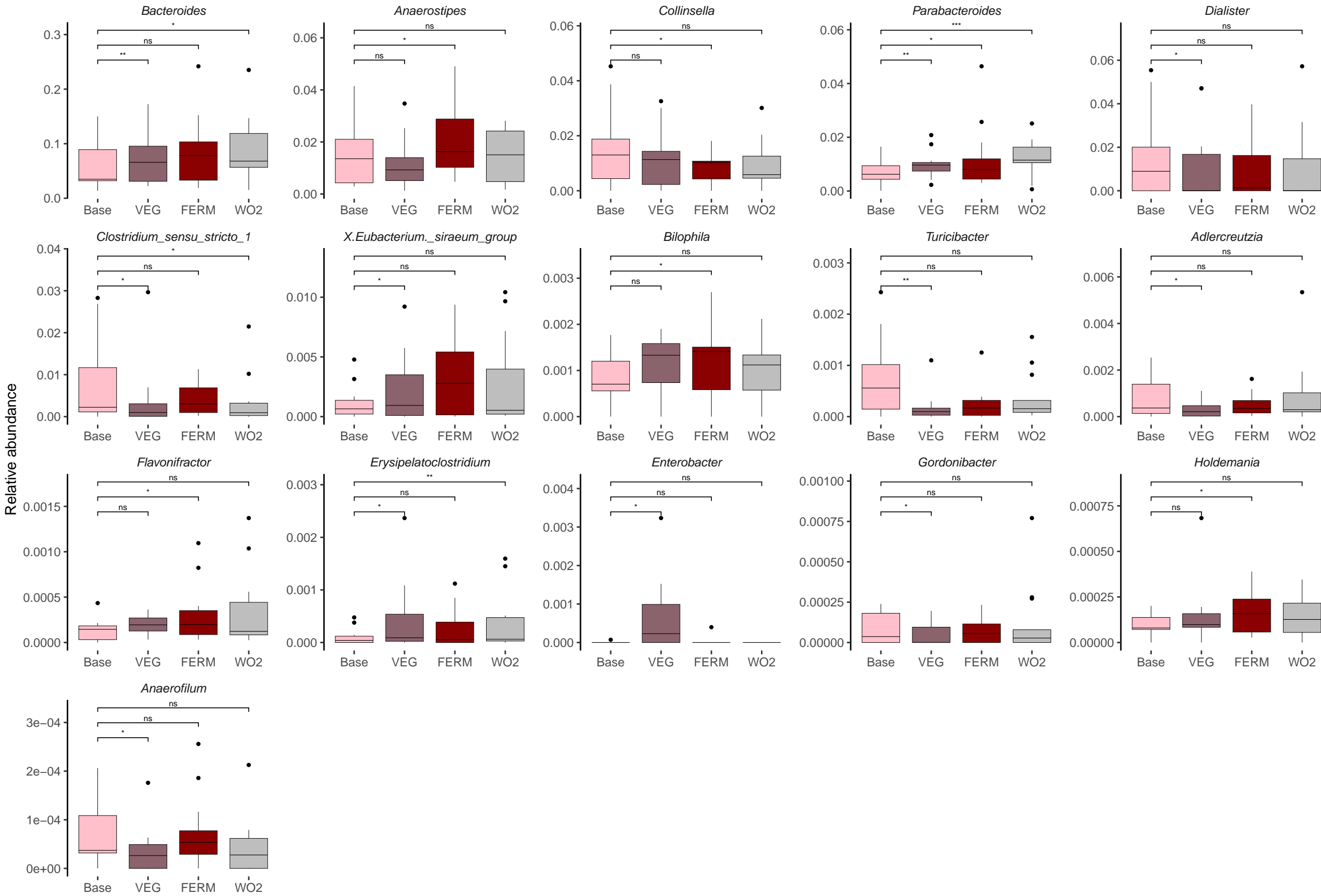

C. HCluster\_3

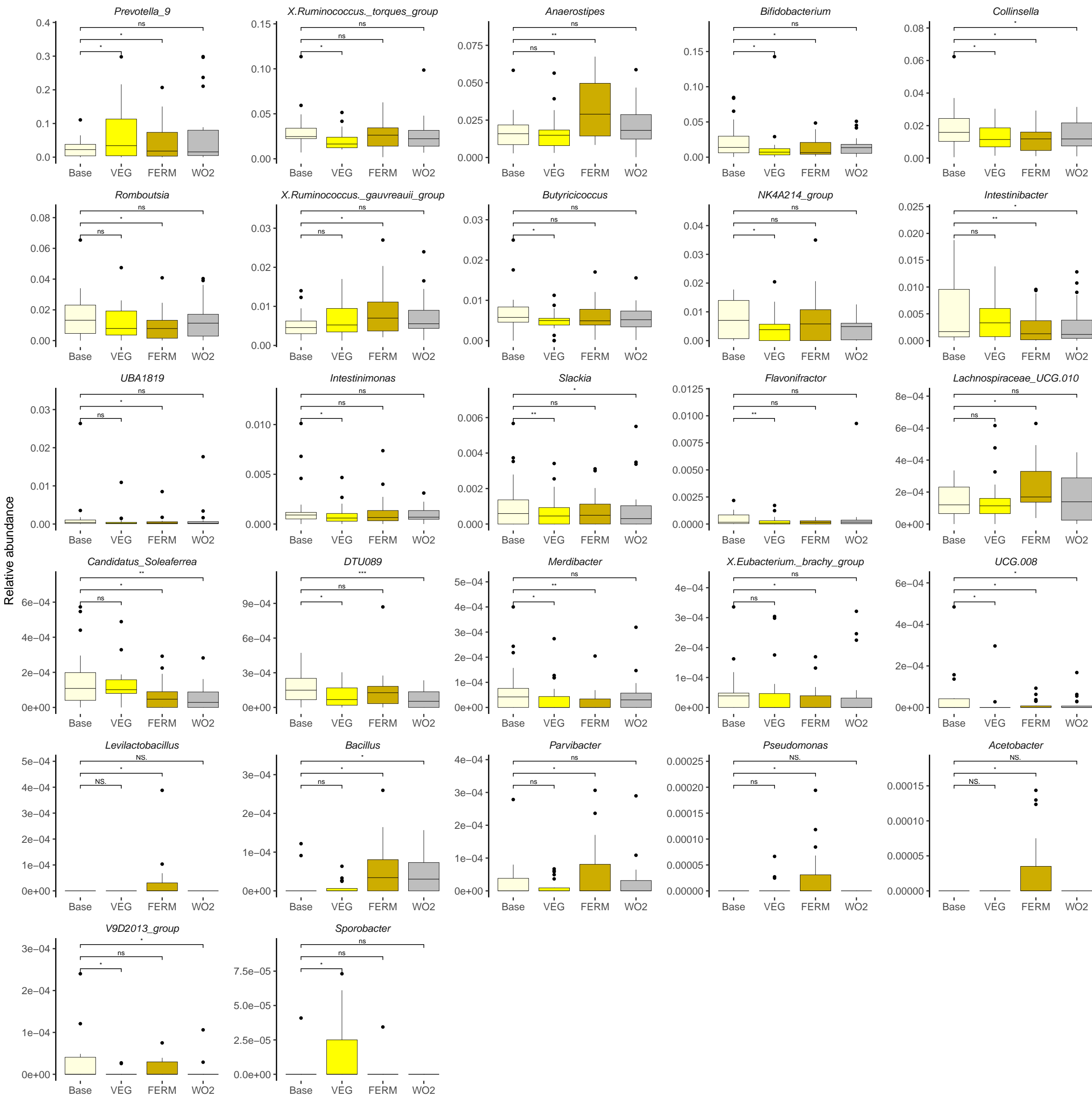

Supplement: Supplementary file 1 [file Supplementary_file_1.zip › Supplementary Figure 6.PDF]

A. Baseline (P1)

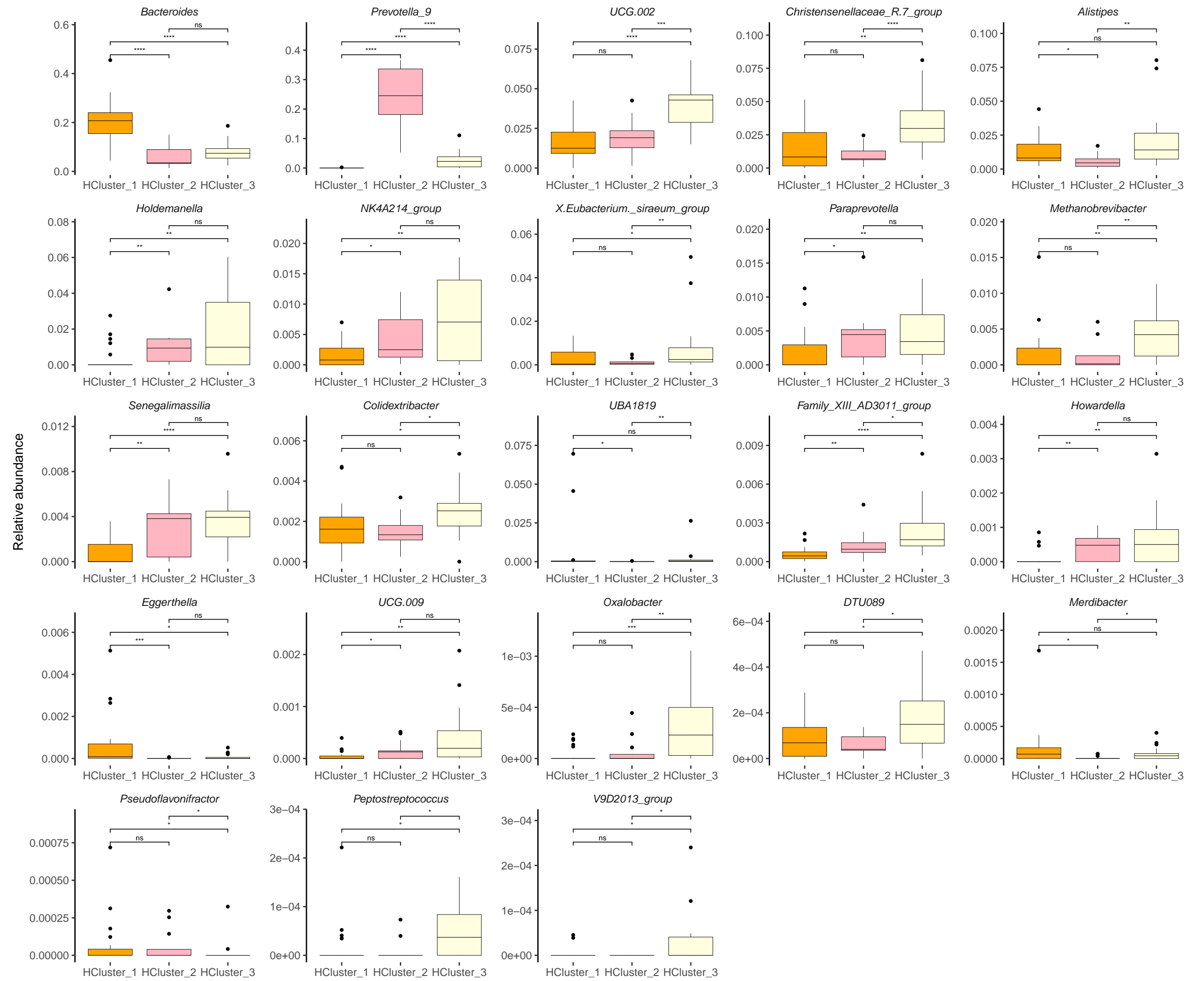

B. End of the study (P5)

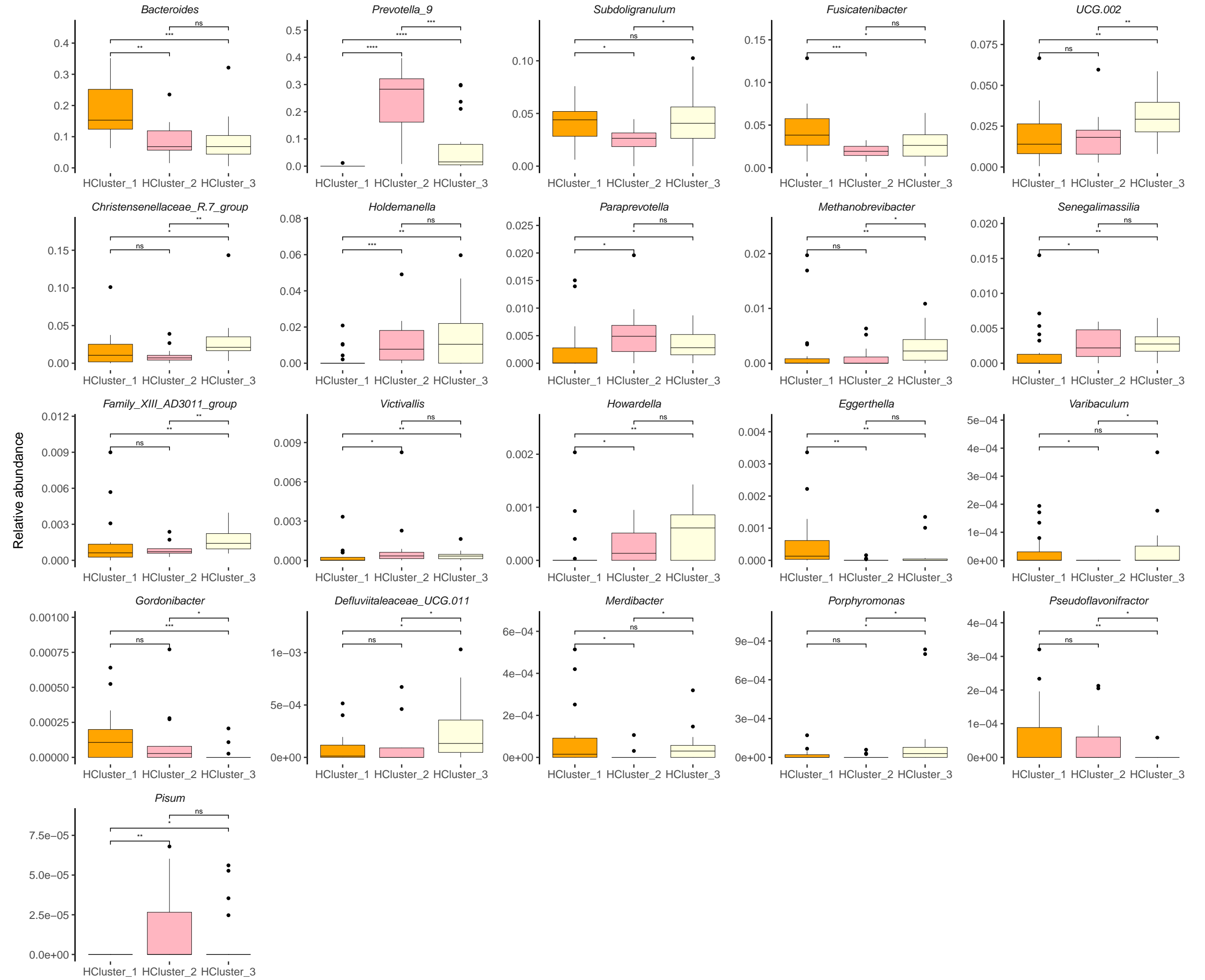

Supplement: Supplementary file 1 [file Supplementary_file_1.zip › Supplementary Figure 4.PDF]

### A. Control group

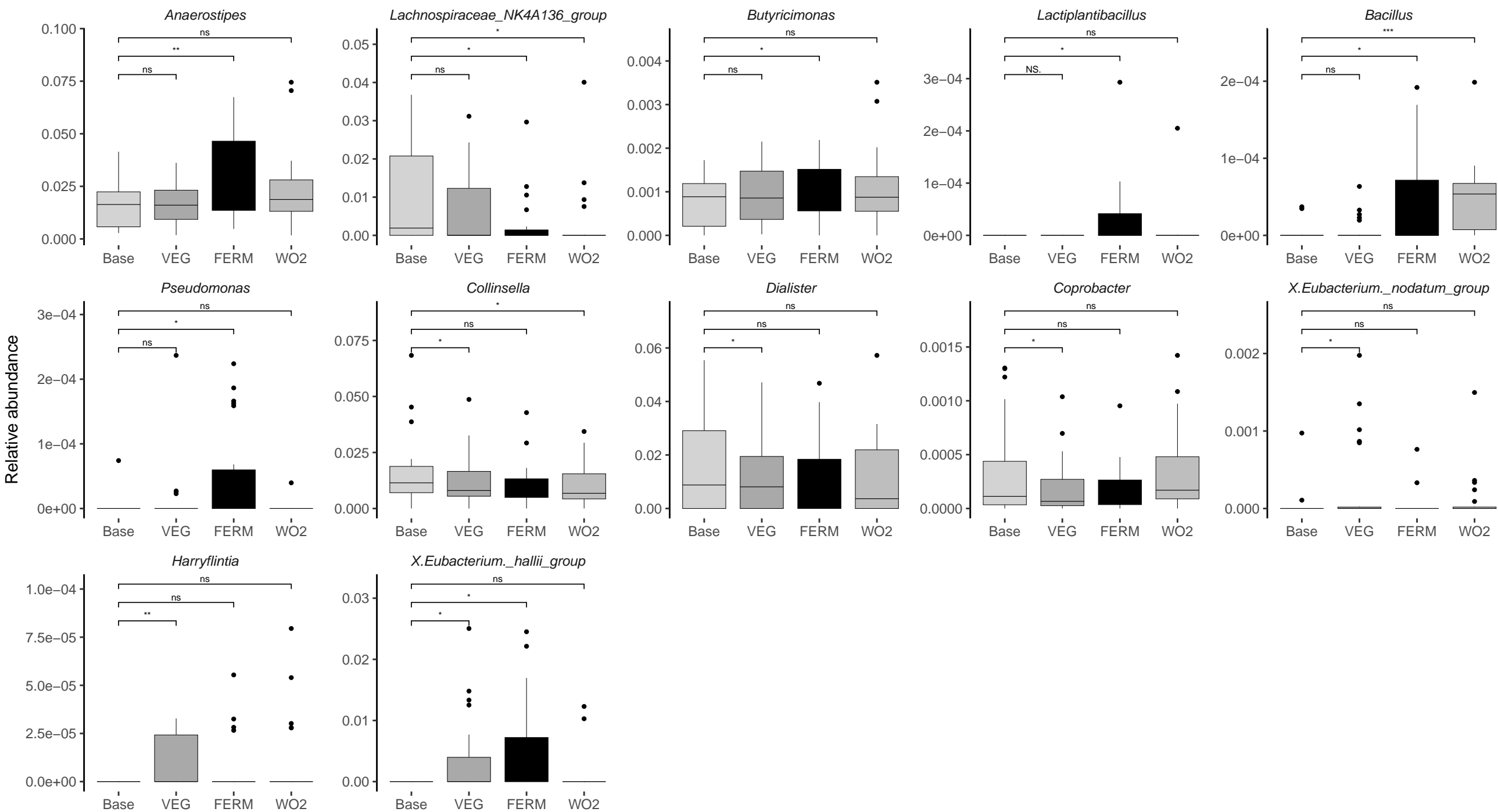

### B. Constipation group

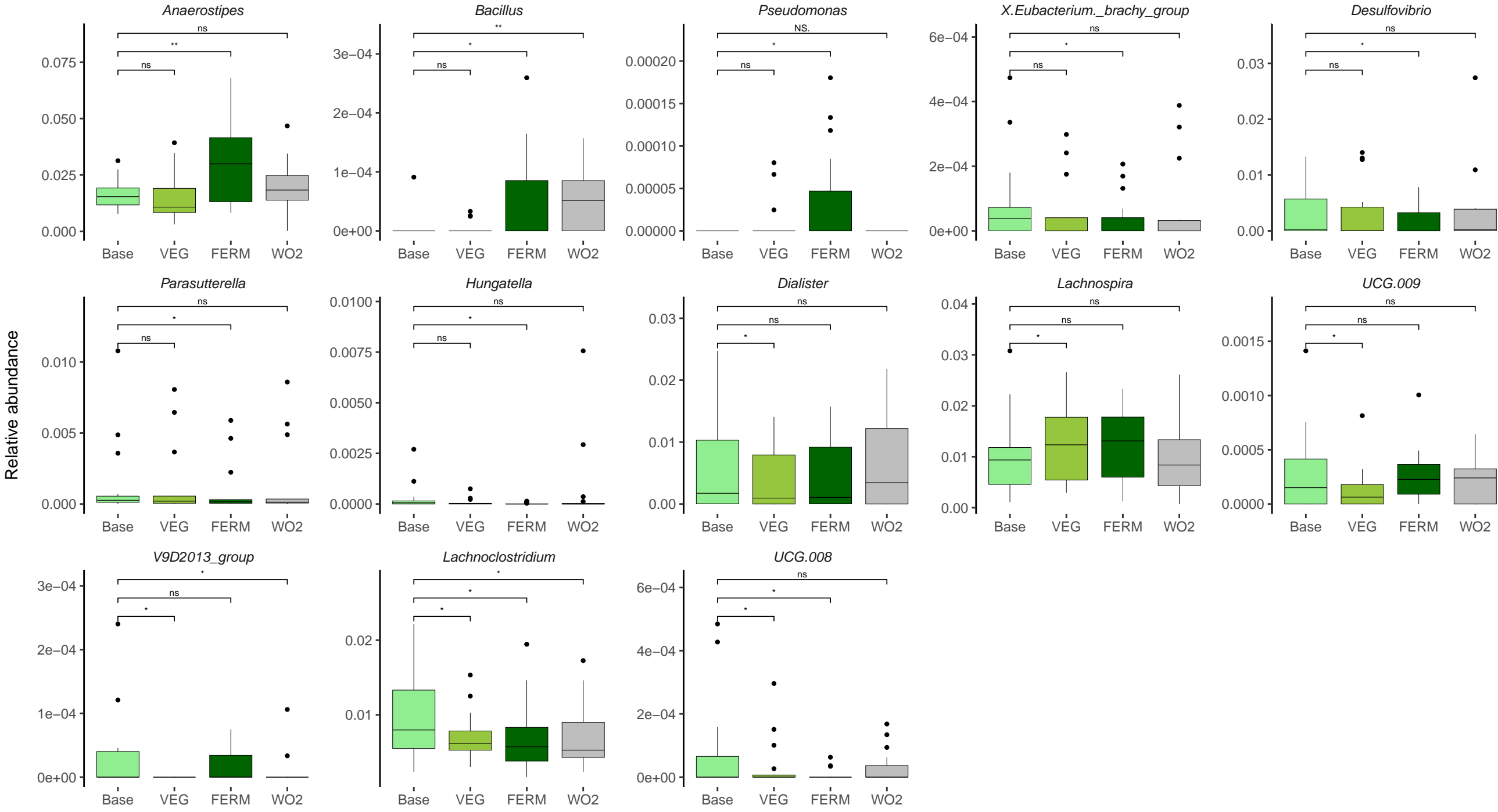

### C. Antibiotics group

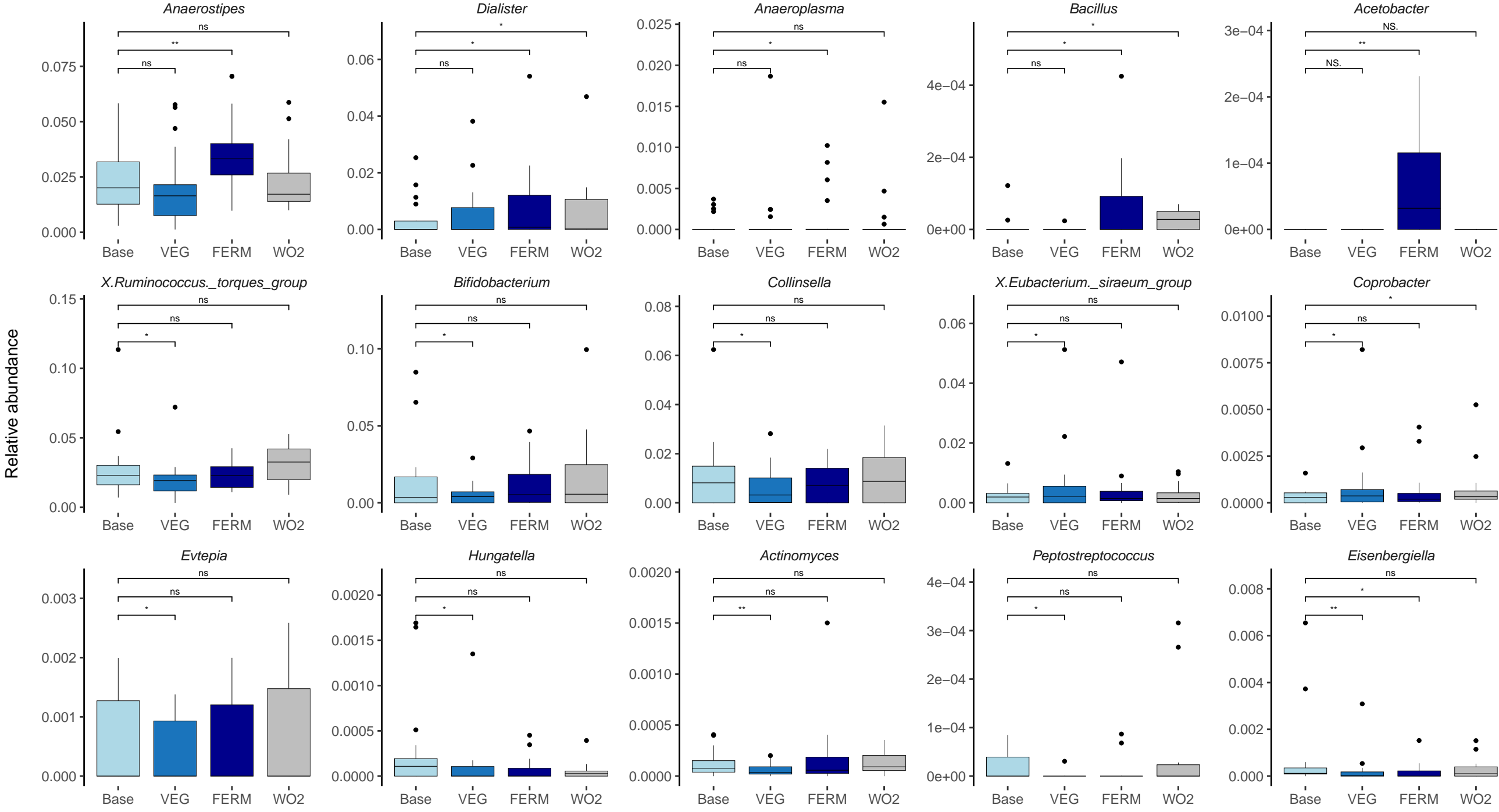

Supplement: Supplementary file 1 [file Supplementary_file_1.zip › Supplementary Figure 3.PDF]

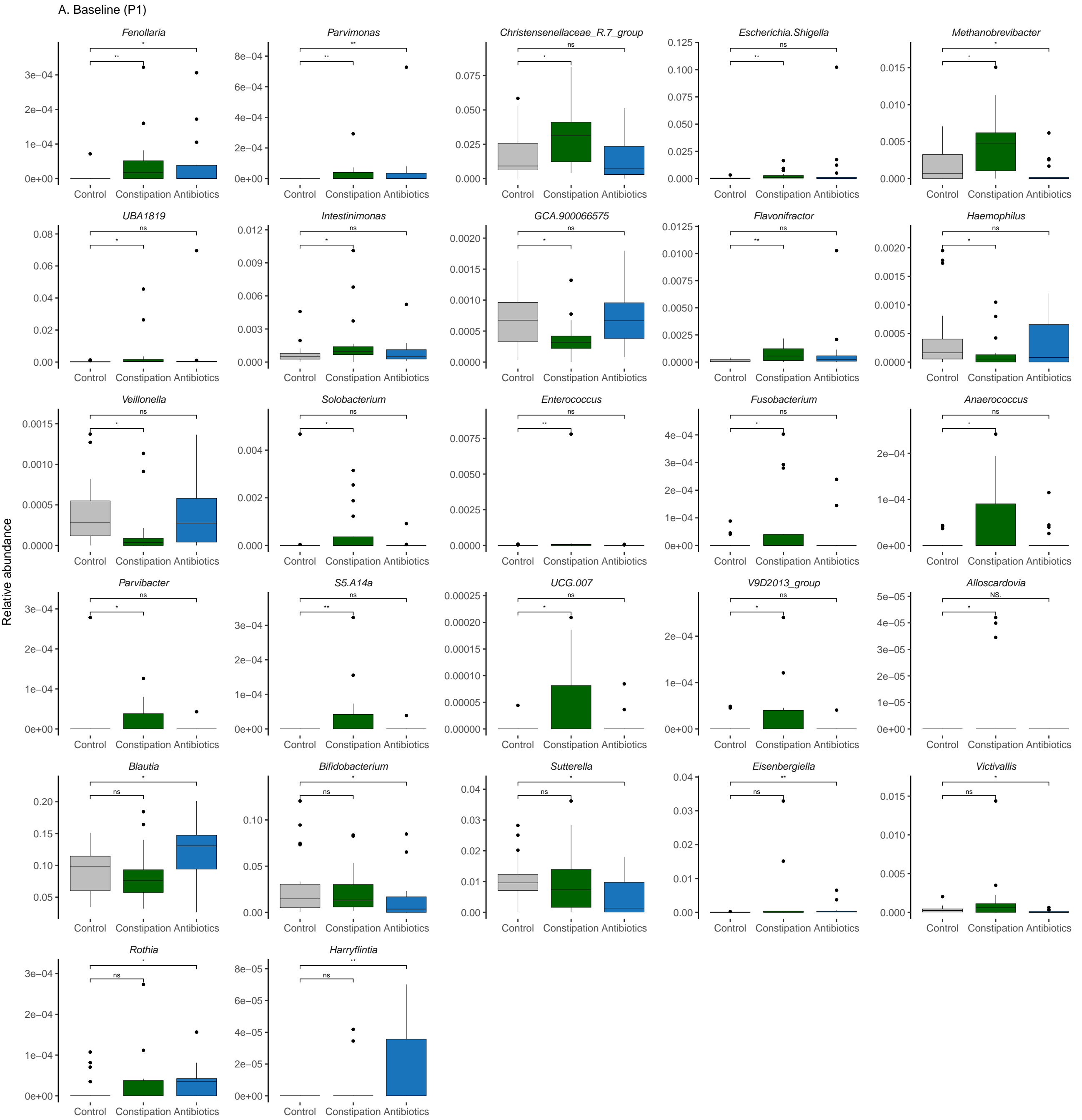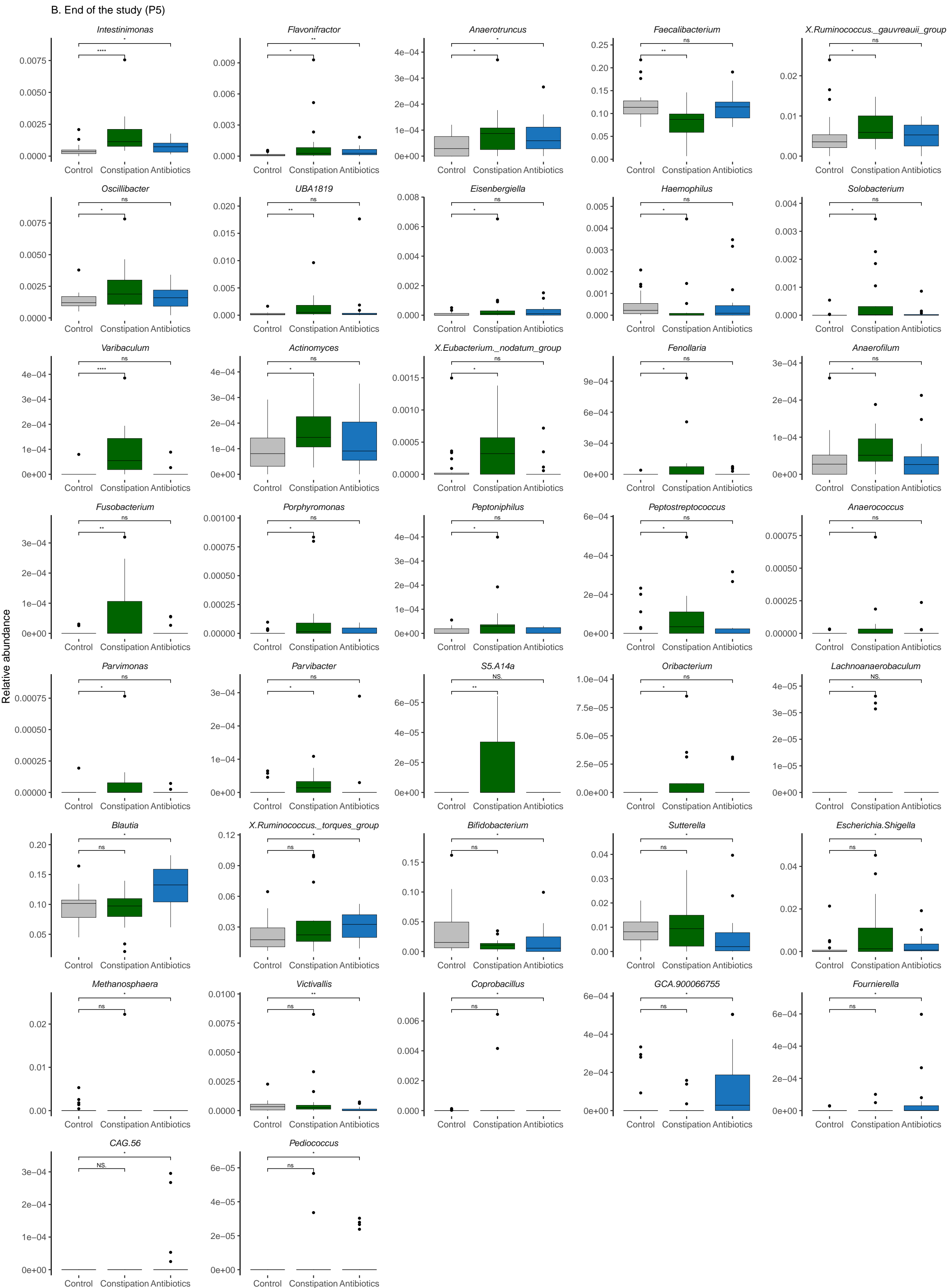

Supplement: Supplementary file 1 [file Supplementary_file_1.zip › Supplementary Figure 1.PDF]
